# Supplementary material for: Mitochondrial genome microhomology-mediated editing by donor DNA delivery into mitochondria in human cells
Source: Mol Ther Nucleic Acids. 2026 May 19;37(2):102959. doi: 10.1016/j.omtn.2026.102959 (PMC13267557; doi:10.1016/j.omtn.2026.102959)
Supplement: Document S1. Figures S1–S3 and Tables S1–S3, S5, and S7–S10 [file mmc1.pdf]

OMTN, Volume *37*

Supplemental information

Mitochondrial genome microhomology-mediated editing by donor DNA delivery into mitochondria in human cells

Vadim V. Maximov, Nikita Shebanov, Natalia Nikitchina, Rachel Rapoport, Yehoshua Maor, Ivan Tarassov, Ophry Pines, and Nina Entelis

Supplemental Material

Table S1. Oligonucleotides Used in the MMEJ Assays. Annealing sites P5 and P7 primers are underlined. The BamHI site in the oligonucleotides MMEJ-Linker-Dir and MMEJ-Linker-Rev is also underlined. Microhomology sites are in italic. All the other nucleotides are in bold.

Oligonucleotide Name	Oligonucleotide Sequence
MMEJ-L-HS-Dir	**GACT**AATGATACGGCGACCACCGA<br>*CACTCACAGTCGCATCATAATC***ATGCATAA**
MMEJ-L-HS-Rev	**GCAT***GATTATGATGCGACTGTGAGTG*<br>TCGGTGGTCGCCGTATCATT
MMEJ-R-HS-Dir	**GATTAGAT***CACTCACAGTCGCATCATAATC*<br>TCGTATGCCGTCTTCTGCTTG**TGAG**
MMEJ-R-HS-Rev	CAAGCAGAAGACGGCATACGA<br>*GATTATGATGCGACTGTGAGTG***ATCT**
MMEJ-L-HS1-Dir	**GACT**AATGATACGGCGACCACCGA<br>*TCAAACTACGAACGCACTCACA***ATGCATAA**
MMEJ-L-HS1-Rev	**GCAT***TGTGAGTGCGTTCGTAGTTTGA*<br>TCGGTGGTCGCCGTATCATT
MMEJ-Linker-Dir	**CACAGTCC***TCAAACTACGAACGCACTCACA*<br>GGATCC*TCATAATCCTCTCTCAAGGACT***AGACGTGC**
MMEJ-Linker-Rev	**GTCT***AGTCCTTGAGAGAGGATTATGA*<br>GGATCC*TGTGAGTGCGTTCGTAGTTTGA***GGAC**

MMEJ-R-HS2-Dir	**GATTAGAT***TCATAATCCTCTCTCAAGGACT*<br>TCGTATGCCGTCTTCTGCTTG**TGAG**
MMEJ-R-HS2-Rev	CAAGCAGAAGACGGCATACGA<br>*AGTCCTTGAGAGAGGATTATGA***ATCT**
MMEJ-Dir	GACTAATGATACGGCGACCACCGATCAAACTACGA<br>ACGCACTCACAGTCGCATCATAATCCTCTCTCAAG<br>GACTTCGTATGCCGTCTTCTGCTTGTGAG
MMEJ-Rev	CAAGCAGAAGACGGCATACGAAGTCCTTGAGAGAG<br>GATTATGATGCGACTGTGAGTGCGTTCGTAGTTTGA<br>TCGGTGGTCGCCGTATCATT

Table S2. Primers used in this study.

**Primer Name**	**Primer Sequence**
P5	AATGATACGGCGACCACCGA
P7	CAAGCAGAAGACGGCATACGA
Mit-ND4-DS-Dir	TCGTCGGCAGCGTCAGATGTGTATAAGAGACAG<br>CCACGGGCTTACATCCTCAT
Mit-ND4-DS-Rev	GTCTCGTGGGCTCGGAGATGTGTATAAGAGACAG<br>GCGAGGCTTGCTAGAAGTCA
Ext Pimer	TCGTCGGCAGCGTCAGAT
NGS-ND4-Dir	TCGTCGGCAGCGTCAGATGTGTATAAGAGACAG<br>NNNNNNNNNNNNGGGGTAAGGCGAGGTTAGC

NGS-ND4-Rev	GTCTCGTGGGCTCGGAGATGTGTATAAGAGACAG CCCTCGTAGTAACAGCCATTC

Table S3. Human Nuclear mitochondrial Sequences (NumtS) similar to the human mitochondrial sequence >chrM:11713-11853.

**Genomic Location 1**	**>chr5:134926943-134927083**
Genomic Sequence 1	GTGAGGCTTGCTAGAAGTCATCAAAAGGCTATTAGTGGGA GTAGGGTTTGAAGTCCTTGAGAGAGAATTATGATGCGACT GTGGGTACGTTCGTAGTTTGAGTTTGCTAGGCAGAATAGTA ATGAGGATGTAAGTCCGTGG
**Genomic Location 2**	**>chr5:100049269-100049409**
Genomic Sequence 2	GCGAGGCTTGCCAGAAGTCATCAAAAGGCTATTAGTGGGAG TAGGGTTTGAAGTCCTTGAGAGAGAATTATGATGCGGCTGT GGGTTCGCTCGTAGTTTGAGTTTGCTAGGCAGAAAAATAAG GAGGATGTAAGTCCGTGG
**Genomic Location 3**	**>chr7:64104212-64104352**
Genomic Sequence 3	GTAAGATTTGCTATAAGTCTTCAAAAGGCTATTAGTGGGAG TAGTGTTTGAAGCTCTCGAGAGAGTAATATGATTCGGCTAT GGATTCGCTCATAGTTTGAATTTGCTAGGCAGAATAGTAAG GACAAAGTAAGTCCATGG
**Genomic Location 4**	**>chr4:155455324-155455464**
Genomic Sequence 4	GTAAGATTTGCTAGAAGTCATCAAGAGGCTATTAGCGGGA

	GCAGTGTTTGAAGGCCTCAGGTAAGAAATATGGTTTGGCTA TGGACTTGCTCGTAGTTTGAATTTGCTAGGCAGAAGAGTAA GGATGAAGTGAGTCCATGA
**Genomic Location 5**	**>chr8:67585876-67586015**
Genomic Sequence 5	GTCTGATTTGCTAGAAGTCATCCTGAGGCTATTAGTGGGAGT AGTGTTTGAAGGCCTCAGTAAGTAATATGGTTCATCTATGGG CTCACTCATAGTTTGAGTTTGCTAGGCAGAATAGTAGGGAT GAAGTGAGTCCATAA
**Genomic Location 6**	**>chr4:25718362-25718490**
Genomic Sequence 6	GTAAGATTTGCTAGAAGAGGCTATTAGTGGGAGCAGTGTTT GAAGCCCTCGGGTAAGTAATATGGTTCATCTATGGACTCGC TCATAGTTTGAATTTGCTAGGCAGAATAGTAAGGATGAAGT GAATGC
**Genomic Location 7**	**>chr16:10721979-10722119**
Genomic Sequence 7	GTAAGATTTGCTAGAAGTCATCAGGAGGCTATTAGTGGAAG CAATGTTTGAAGGCCTTGGAGAAGTAGTATAATTCGGCTAT GGACTCGTTCATAGTTCCAATTTGCTAGGCAGAATAGTAAG GATGAAGTGAGTCCATGG
**Genomic Location 8**	**>chr1:235540322-235540462**
Genomic Sequence 8	GTAAGATTTGCTAGAAGTCATCAAGAGGCTATTAGCAGAAG CAGTGTTTGAAGGCGTTCGGTAAGTAATAGGGTTCACCTAT GGGCCTGCTCGTAGTTTGAATTTGCTAGGCAGAATAGTAAG GCTGAAGTGAGTCCATGA

Table S4. Numbers of, CPMs of, proportions of, and percentages of reads in the mitochondrial genome editing experiment with donor DNA delivery only. This table can be found in a separate Excel spreadsheet “Table S4.xlsx”.

Table S5. Human Nuclear mitochondrial Sequences (NumtS) similar to the human mitochondrial sequence >chrM:11643-11870.

**Genomic Location 1**	**>chr5:134926926-134927153**
Genomic Sequence 1	CCCTCGTAGTAACAGCCATCCTCATCCAAACCCCCTGAAGC TTCACCGGCGCAGTCATTCTCATAGTCGCCCACGGACTTAC ATCCTCATTACTATTCTGCCTAGCAAACTCAAACTACGAAC GTACCCACAGTCGCATCATAATTCTCTCTCAAGGACTTCAAA CCCTACTCCCACTAATAGCCTTTTGATGACTTCTAGCAAGCC TCACTAATCTCGCCTTACCCC
**Genomic Location 2**	**>chr5:100049260-100049479**
Genomic Sequence 2	CCCTCATAGTAACAGCTATTCTCATCCAAACCCCCTGAAGCT TCACCGGCGCAGTCATTCTCATAATTGCCCACGGACTTACAT CCTCCTTATTTTTCTGCCTAGCAAACTCAAACTACGAGCGAA CCCACAGCCGCATCATAATTCTCTCTCAAGGACTTCAAACCC TACTCCCACTAATAGCCTTTTGATGACTTCTGGCAAGCCTCG CCAACCTCGC
**Genomic Location 3**	**>chr7:64104195-64104401**
Genomic Sequence 3	TCATTCAAACCCCCTGAAGCTTTACTGGTGCAATTACCCTCA

	TAATTGCCCATGGACTTACTTTGTCCTTACTATTCTGCCTAG CAAATTCAAACTATGAGCGAATCCATAGCCGAATCATATTA CTCTCTCGAGAGCTTCAAACACTACTCCCACTAATAGCCTTT TGAAGACTTATAGCAAATCTTACTAACCTTGCCTTACCCC
**Genomic Location 4**	**>chr10:36434949-36435150**
Genomic Sequence 4	CAAACCCCCTGAAGCTTTACTGGTGCAATTACCCTCATAATT GCCCACAGACTTACTCTGTCCTTACTATTCTGCTTAGCAAAT TCAAATTACGAGTGAGTCCACAGCCGAATTATACTGCTCTCT TAACGCCTTCAACCATTACTTCCACTAATAGCTTTTTGATGA CTTACAGCAAATCTTACCAACCTTGCCTTACCCC

Table S6. Numbers of, CPMs of, proportions of, and percentages of reads in the mitochondrial genome editing experiment with donor DNA delivery and CRISPR/mito-AsCas12a. This table can be found in a separate Excel spreadsheet “Table S6.xlsx”.

Table S7. Oligonucleotides used in the oligonucleotide delivery and mitochondrial genome editing assays. Uppercase letters, which represent ribonucleotide residues, are always preceded by a lowercase letter “r”. All the other uppercase letters represent deoxyribonucleotide residues. Deoxyribonucleotide residues, which compose microhomology arms, are in italic. Deoxyribonucleotide residues, which differ from the human mitochondrial genome sequence, are in bold and underlined. Ribonucleotide residues of the crRNA, which are not complementary to the targeted site, are in bold.

Oligonucleotide Name	Oligonucleotide Sequence
RMIS-Dir	rGrCrGrCrArArUrCrGrGrUrArGrCrGrCCAAACTACGAACGCACTCACAGGATCCTCATAATCCTCTCTCAAGGACT
RMIS-Rev	rGrCrGrCrArArUrCrGrGrUrArGrCrGrCAGTCCTTGAGAGAGGATTATGAGGATCCTGTGAGTGCGTTCGTAGTTTG
Dir	CAAACTACGAACGCACTCACAGGATCCTCATAATCCTCTCTCAAGGACT
Rev	AGTCCTTGAGAGAGGATTATGAGGATCCTGTGAGTGCGTTCGTAGTTTG
RMIS-Dir-New	rGrCrGrCrArArUrCrGrGrUrArGrCrGrCCACTCACAGTCGCATCATAATTCTATCACAAGGCCTCCAAACTCTACTCCC
RMIS-Rev-New	rGrCrGrCrArArUrCrGrGrUrArGrCrGrCGGGAGTAGAGTTTGGAGGCCTTGTGATAGAATTATGATGCGACTGTGAGTG
crRNA	rUrArArUrUrUrCrUrArCrUrCrUrUrGrUrArGrArUrArArGrUrCrCrUrUrGrArGrArGrArGrGrArUrUrA
siRNA MGME1 Direct Strand	rGrGrGrUrGrArArArGrUrArUrGrCrUrUrUrCrCrArArGrGrCrUrUrC
siRNA MGME1 Reverse Strand	rArGrCrCrUrUrGrGrArArArGrCrArUrArCrUrUrUrCrArCrCrC

Table S8. Probes for small RNA/DNA Northern blot hybridization.

**Probe Name**	**Probe Sequence**
Anti-tRNA-Thr	TCTCCGGTTTACAAGAC
Anti-5-8S RNA	GGCCGCAAGTGCGTTCGAAG

Table S9. Description of samples for deep sequencing from the mitochondrial genome editing experiment with donor DNA only.

Sample final name	Biological description	Index1	Index2
Sample_1	Mock Transfection	AAGAGGCA	TCGCATAA
Sample_2	Transfection 1	GCTCATGA	ATAGCCTT
Sample_3	Transfection 2	ACTCGCTA	TCTTACGC
Sample_4	Transfection 3	GCGTAGTA	AGCTAGAA
Sample_5	Transfection 4	TACGCTGC	CGGAGAGA

Table S10. Description of samples for deep sequencing from the mitochondrial genome editing experiment with donor DNA and CRISPR/mito-AsCas12a.

Sample final short name	Biological condition	Index 1	Index 2
Sample_CRISPR_1-1	CRISPR + MGME1 siRNA	ACCAATTC	ACTAGAGT
Sample_CRISPR_1-2	CRISPR + MGME1 siRNA	GGTGGACG	CATGAATG
Sample_CRISPR_1-3	CRISPR + MGME1 siRNA	TGCGTCTC	GTTCATAG
Sample_CRISPR_2-1	ssDNA + CRISPR	GGATTGTG	GTGGATAT
Sample_CRISPR_2-2	ssDNA + CRISPR	GTTGCCGG	TTAAACCG
Sample_CRISPR_2-3	ssDNA + CRISPR	CGTAAAGT	GGCGTTCA
Sample_CRISPR_3-1	ssDNA + CRISPR + MGME1 siRNA	GGTGTGAA	GTACACTA
Sample_CRISPR_3-2	ssDNA + CRISPR + MGME1 siRNA	AGGTTATC	TTCTAATG
Sample_CRISPR_3-3	ssDNA + CRISPR + MGME1 siRNA	TCGATTGT	TTCCGTCA
Sample_CRISPR_4-1	dsDNA	CTAGATAA	GAGCCTTT
Sample_CRISPR_4-2	dsDNA	GGGTTCCG	CTCGAATC
Sample_CRISPR_4-3	dsDNA	AAGTAAGA	AGAGGTCA
Sample_CRISPR_5-1	dsDNA + CRISPR	TCACCCAT	TTCCCAAG

Sample_CRISPR_5-2	dsDNA + CRISPR	AATATTCG	GCTTCCAG
Sample_CRISPR_5-3	dsDNA + CRISPR	GTCCCGGA	GCATTCAC
Sample_CRISPR_6-1	dsDNA + CRISPR + MGME1 siRNA	GTGCGGTG	TGACAGTC
Sample_CRISPR_6-2	dsDNA + CRISPR + MGME1 siRNA	CCTTGACG	GTGACCTA
Sample_CRISPR_6-3	dsDNA + CRISPR + MGME1 siRNA	CTTGACGA	TGAGTTGC

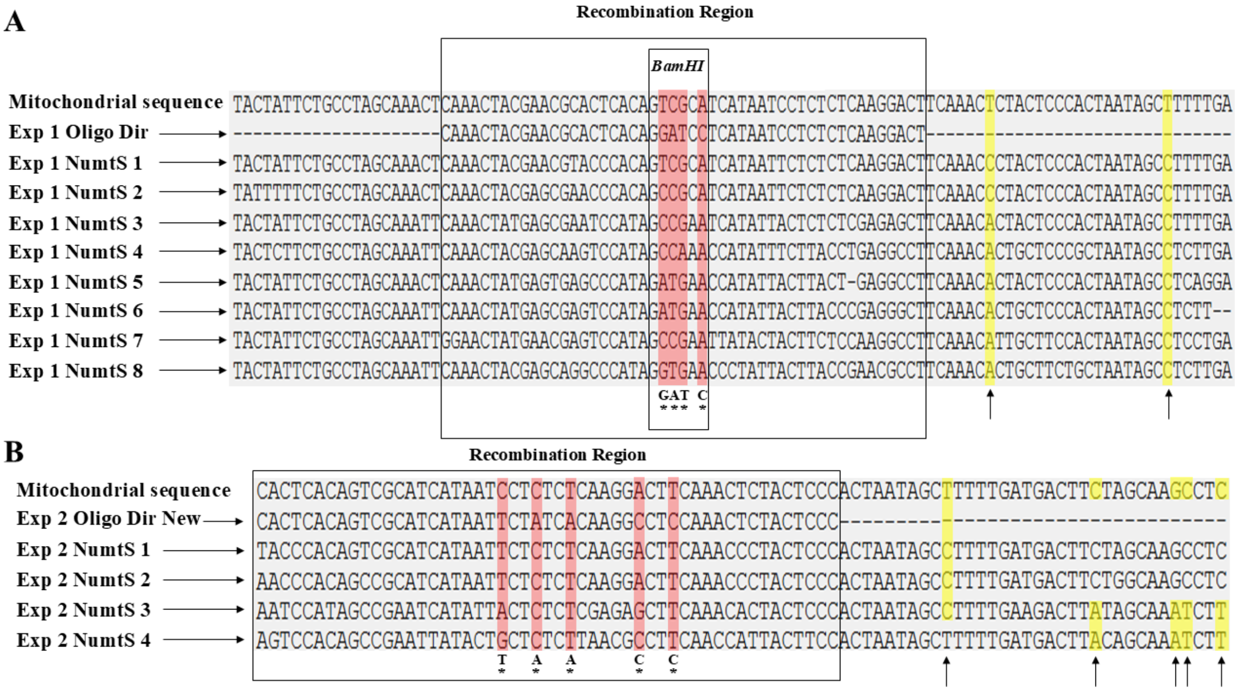

Figure S1. Alignment of the amplified human mitochondrial sequences to similar nuclear mitochondrial sequences (NumtS) at the sequenced amplicon region encompassing the edited *ND4* site. Alignments of a part the amplified and sequenced human mitochondrial genomic region,

harboring the edited *ND4* site, to the donor DNA sequences (Dir and Dir New) with the expected nucleotide edits, as well as to corresponding human NumtS, are shown. The alignments were conducted by the MAFFT software (https://mafft.cbrc.jp/alignment/server/index.html)[1,2]. Primers annealing sites for the amplified mitochondrial sequence and corresponding nuclear sequences are not included in the figure. The expected nucleotide edits are indicated at the bottom in bold and marked with asterisks. Corresponding nucleotides in the aligned sequences are highlighted in pink. The human nuclear sequences with the primers annealing sites can be found in Supplemental Tables 5 and 6. The recombination regions are surrounded by large rectangles. Nucleotide positions outside the recombination regions, at which the mitochondrial sequences differ from the corresponding nuclear sequences, are highlighted in yellow and marked by arrows at the bottom. (A) Alignment of the entire amplified and sequenced human mitochondrial genomic sequence without primers annealing sites from the experiment presented on Figure 3 to corresponding donor DNA and human NumtS is shown. The small rectangle surrounds sequence, which corresponds to the BamHI site in the edited mitochondrial DNA. (B) Alignment of the right part of the amplified and sequenced human mitochondrial genomic sequence without the primer annealing site from the experiment presented on Figure 4 to corresponding donor DNA and human NumtS is shown.

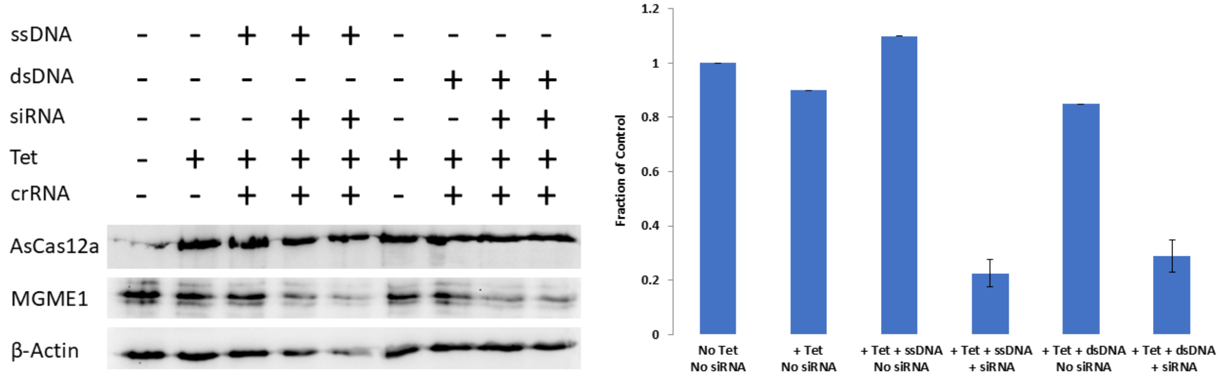

Figure S2. Western blot data on MGME1 and AsCpf1 expression under various experimental conditions. (Left panels) Western blot images are shown. (The right panel) Quantification of the MGME1 western blot bands with normalization to *β*-actin is shown. Data are presented as fraction of control without any treatment. "ssDNA" indicates transfections with the RMIS-Rev-New oligonucleotide. "dsDNA" indicates transfections with the RMIS-Dir-New:RMIS-Rev-New oligonucleotide duplex. "siRNA" indicates transfections with the *MGME1* siRNA. "Tet" indicates addition of tetracycline to the culturing medium for the *mito-AsCas12a* expression induction. "crRNA" indicates crRNA, which targets the mitochondrial *ND4* gene at the edited mtDNA site.

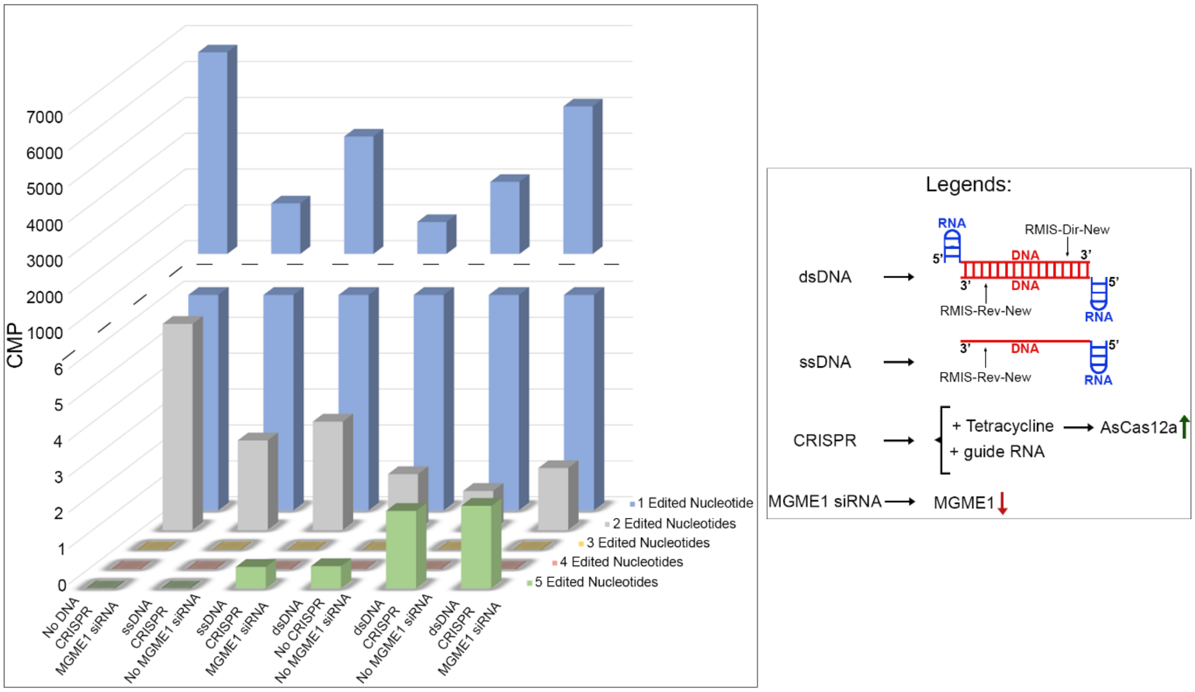

Figure S3. Introduction of changes in mtDNA by donor DNA delivery and CRISPR in mitochondria of T-REx-293-Su9-AsCas12a cells – supplemental information. Left panel: counts of reads with five-, four-, three-, two-, or one-edited nucleotides per one million (Counts Per Million = CPM) of reads unambiguously aligned to the mitochondrial sequence (MT:11643-11870) for each transfection are shown on the 3D graph. Right panel: legends, explaining transfections and treatments.

Supplemental References.

1. Katoh, K., Rozewicki, J. & Yamada, K. D. MAFFT online service: multiple sequence alignment, interactive sequence choice and visualization. *Brief Bioinform* **20**, 1160–1166 (2019).

2. Kuraku, S., Zmasek, C. M., Nishimura, O. & Katoh, K. aLeaves facilitates on-demand exploration of metazoan gene family trees on MAFFT sequence alignment server with enhanced interactivity. *Nucleic Acids Res* **41**, W22–W28 (2013).
