# Supplementary material for: Mitochondrial genome microhomology-mediated editing by donor DNA delivery into mitochondria in human cells
Source: Mol Ther Nucleic Acids. 2026 May 19;37(2):102959. doi: 10.1016/j.omtn.2026.102959 (PMC13267557; doi:10.1016/j.omtn.2026.102959)
Supplement: Video abstract [file mmc2.pdf]

Video Abstract Transcript.

A human cell harbors from hundreds to thousands of mitochondria. Each human mitochondrion contains several copies of mitochondrial DNA vulnerable to mutations, which can be pathogenic. Multiplication of mitochondrial DNA molecules with pathogenic mutations attenuates mitochondrial functions, leading to a mitochondrial disease. We may expect that correction of mitochondrial DNA pathogenic mutations may cure mitochondrial pathologies.

Cytosine and adenosine deaminases-based tools have been developed during this decade to introduce changes in human mitochondrial DNA and correct pathogenic mitochondrial DNA mutations. These enzymes recognize and deaminate cytosines or adenines in chosen positions and produce uracils or inosine bases, which are, after mitochondrial DNA replication, converted into thymines or guanines, correspondingly. However, the available tools have a fundamental limitation—they introduce only point transition nucleotide changes. In other words, deaminases-based tools can convert a purine base into the other purine base and a pyrimidine base into the other pyrimidine base.

We developed an alternative method of introducing changes in the human mitochondrial DNA sequence. This method can introduce any changes in mitochondrial DNA. Our method is based on delivery of donor DNA in mitochondrial matrix and microhomology-mediated site-specific integration of the donor DNA in mitochondrial DNA with replacement of the homologues host sequence. We named this method: microhomology-mediated editing of mitochondrial DNA.

Firstly, we proved that enzymatic activities of human mitochondrial extracts permit microhomology-mediated ends joining. Secondly, we found that an RNA mitochondrial import signal can be used to target double-stranded DNA duplexes in mitochondrial matrix. Thirdly, we tested whether mitochondria-delivered donor DNA with microhomology arms can integrate in intact or CRISPR-cleaved mitochondrial DNA and replace the host DNA between microhomology sites, thus introducing changes in several nucleotides of the mitochondrial DNA in live human cells. We found low but statistically significant proportion of pre-designed nucleotide changes in mitochondrial DNA.

Overall, the developed method of introducing changes in mitochondrial DNA opens avenue to new approaches of gene therapy of primary mitochondrial diseases, which are caused by mutations in mitochondrial DNA.
