# Supplementary material for: Mitochondrial genome microhomology-mediated editing by donor DNA delivery into mitochondria in human cells
Source: Mol Ther Nucleic Acids. 2026 May 19;37(2):102959. doi: 10.1016/j.omtn.2026.102959 (PMC13267557; doi:10.1016/j.omtn.2026.102959)
Supplement: Document S2. Article plus supplemental information [file mmc6.pdf]

Molecular Therapy
Nucleic Acids
Original Article

Mitochondrial genome microhomology-mediated editing by donor DNA delivery into mitochondria in human cells

Vadim V. Maximov,[1,3] Nikita Shebanov,[2] Natalia Nikitchina,[2] Rachel Rapoport,[3] Yehoshua Maor,[1] Ivan Tarassov,[2] Ophry Pines,[3] and Nina Entelis[2]

[1]Phytor Ltd, Jerusalem 9726313, Israel; [2]UMR7156 Molecular Genetics, Genomics, Microbiology, CNRS/University of Strasbourg, 67000 Strasbourg, France; [3]Department of Microbiology and Molecular Genetics, IMRIC, The Hebrew University of Jerusalem, Jerusalem 9112102, Israel

Mutations in mitochondrial DNA (mtDNA) are associated with severe human diseases, lacking efficient therapies. Direct correction of mtDNA mutations may offer a cure for such diseases. We propose a novel strategy based on double-stranded DNA (dsDNA) oligonucleotide delivery into mitochondria and intrinsic microhomology-mediated end joining (MMEJ) for mtDNA editing. This strategy enables the introduction of multiple predefined nucleotide changes in mtDNA. For this, the presence of MMEJ activity in the human mitochondrial lysates was confirmed. Forty-nine bp DNA oligonucleotide duplexes, fused to an RNA hairpin previously identified as a mitochondrial import signal, were delivered into the mitochondria of cultured human cells. Delivery of these donor dsDNA molecules, homologous to an *ND4* site of mtDNA and bearing designed nucleotide changes, led to a low but statistically significant introduction of the intended nucleotide changes into mtDNA. Donor dsDNA delivery combined with the CRISPR-mito-AsCas12a system also resulted in a statistically significant number of an expected concomitant change of five nucleotides distributed across a 16 nt *ND4* site of the mitochondrial genome. The proposed strategy may become an efficient mtDNA editing tool suitable for the correction of near-homoplasmic mutations, such as Leber's hereditary optic neuropathy (LHON)-associated mutations in the *ND4* gene of mtDNA.

INTRODUCTION

Mutations in mitochondrial DNA (mtDNA) are associated with a spectrum of health conditions, including cancer, aging, and Parkinson's disease,[1,2] in addition to their roles in primary mitochondrial diseases.[3,4] Leber's hereditary optic neuropathy (LHON), which causes central vision loss at a young age, is the most common optic neuropathy and an important example of a mitochondrial disease.[5] The most frequent mutation 11,778G>A, which is associated with LHON, occurs in the mitochondrial NADH dehydrogenase subunit 4 (*ND4*) gene[6,7] (Figure 1A), which is a component of the mitochondrial respiratory chain.[8] This mutation leads to R340H substitution in the ND4 protein[6] (Figure 1A). Functional studies of disease-associated mtDNA mutations, as well as the study of the mammalian mitochondrial genome in general, were impeded by the lack of efficient mammalian mitochondrial genome editing tools. Such tools could also be applied for gene therapies against diseases caused by mutations in the mtDNA.

Cytosine and adenosine deaminases-based tools have enabled the introduction of point transition nucleotide substitutions in mammalian mtDNA. Mitochondrially targeted rat APOBEC1, which can act as an RNA and DNA cytosine deaminase, was initially applied to introduce random cytidine to thymidine transition mutations in the Drosophila mitochondrial genome.[9] The first prominent success in mammalian mitochondrial genome editing was achieved by applying the double-stranded DNA (dsDNA) deaminase toxin A (DddA)-derived cytosine base editor (CBE) (DdCBE).[10–19] Mitochondrial adenosine base editors (ABEs) for efficient A-to-G editing, which are known as TALE-linked deaminases (TALEDs), have also been developed. TALEDs are fusions of TALE domains with a modified *E. coli* deoxyadenosine deaminase TadA—TadA8e.[18,20,21] Despite these impressive achievements, deaminase-based techniques have a fundamental limitation—they can introduce only point transition nucleotide changes (purine-to-purine or pyrimidine-to-pyrimidine). All other modifications of the mammalian mitochondrial genome—transversion nucleotide changes (purine-to-pyrimidine or pyrimidine-to-purine), deletions, insertions, inversions, etc.—remain a challenge and require the development of novel mitochondrial genome editing strategies.

Received 7 November 2025; accepted 15 May 2026;
https://doi.org/10.1016/j.omtn.2026.102959.

Correspondence: Vadim V. Maximov, Department of Microbiology and Molecular Genetics, HUJ and Phytor Ltd, Jerusalem 9726313, Israel.
E-mail: vadim.phytor@gmail.com
Correspondence: Ivan Tarassov, UMR7156 Molecular Genetics, Genomics, Microbiology, CNRS/University of Strasbourg, 4, Allée Konrad Roentgen, Strasbourg 67000, France.
E-mail: i.tarassov@unistra.fr

© 2026 The Author(s).
Published by Elsevier Inc. on behalf of The American Society of Gene and Cell Therapy.
This is an open access article under the CC BY license (http://creativecommons.org/licenses/by/4.0/).

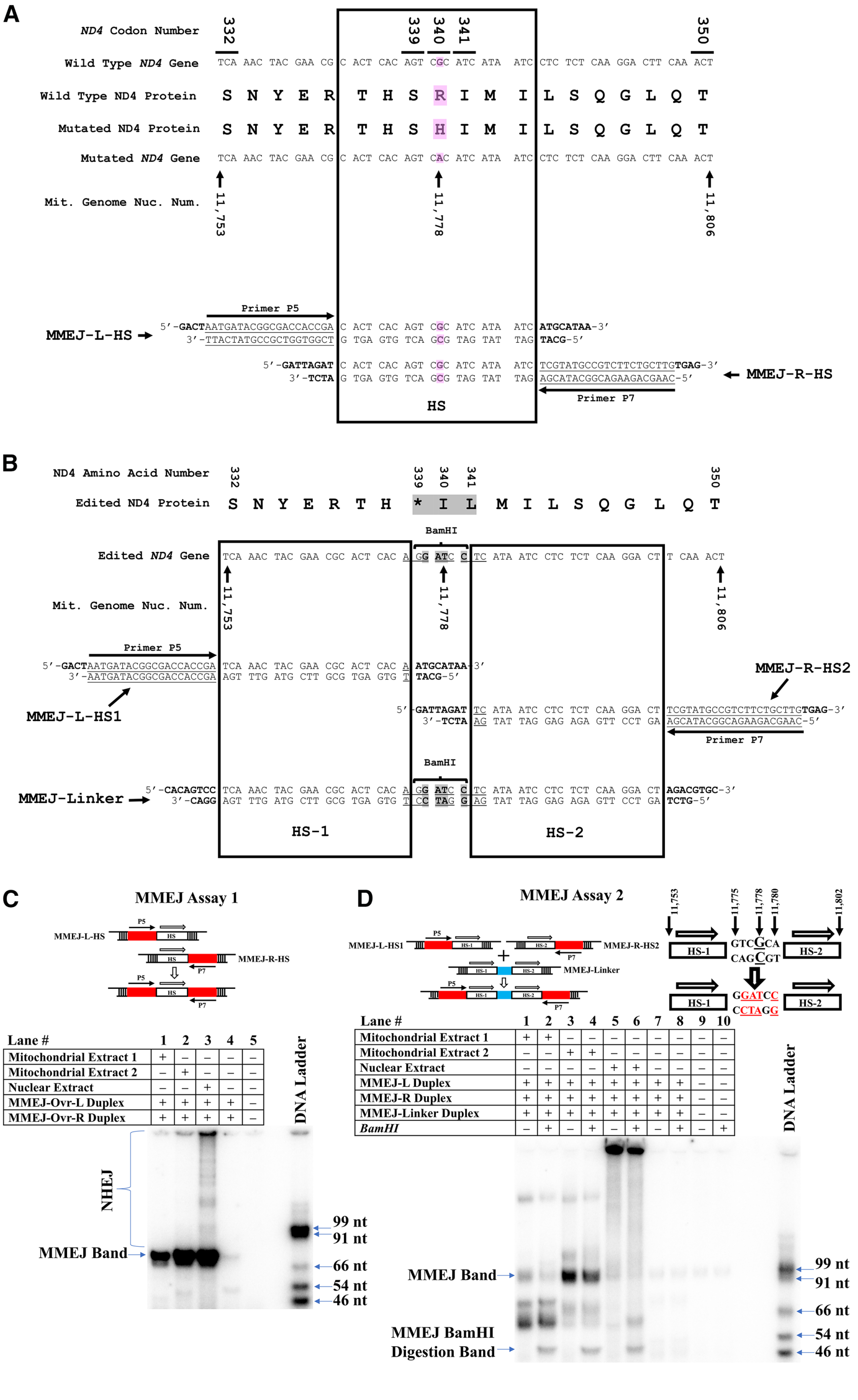

Figure 1. MMEJ Assay

(A) Sequences of the wild-type and LHON-associated mutant *ND4* gene alleles and ND4 proteins (upper) aligned to MMEJ-L-HS and MMEJ-R-HS duplexes (lower). Nucleotides and amino acid residues, which differ between wild-type and mutant alleles and proteins, are highlighted in purple. Sequences within the HS rectangle corresponds to the microhomology recombination site HS. (B) DNA duplexes applied for BamHI site insertion by MMJE (lower) and changes introduced in the *ND4* gene and ND4 protein during the mitochondrial genome editing by donor DNA delivery alone. The edited *ND4* gene nucleotides and corresponding amino acids are highlighted in bold gray. Sequences in the HS-1 and HS-2 rectangles correspond to the microhomology recombination sites HS-1 and HS-2. (C) End-joining activity in mitochondrial and nuclear extracts tested with DNA duplexes MMEJ-L-HS and MMEJ-R-HS as schematically represented at the top. The MMEJ product was detected by autoradiography of fragments obtained by PCR with P5 and ^{32}P-labeled P7 primers (highlighted in red) and separated by 10% urea PAGE. The HS sequence is shown in (A). The positions of MMEJ and NHEJ products are indicated. (D) End-joining activity is tested in mitochondrial and nuclear extracts in the presence of three DNA duplexes: MMEJ-L-HS1, MMEJ-linker, and MMEJ-R-HS2, as schematically shown. BamHI site is shown in blue. HS-1 and HS-2 sequences are shown in (B). Nucleotide G11778, which is commonly mutated in LHON, is underlined and enlarged. Nucleotides, which substitute the natural ones for the creation of the BamHI site, are highlighted in red and underlined. Autoradiography of MMEJ PCR product and its fragment (MMEJ BamHI digestion band) separated by 10% urea PAGE, is shown. DNA ladder represents a mix of ^{32}P-labeled oligonucleotides, and the size of each band is indicated at the right.

Mammalian mitochondria lack the classical non-homologous end joining (NHEJ),[22] and linearized mtDNA is typically degraded by components of the replication machinery.[23,24] Remarkably, microhomology-mediated end joining (MMEJ), as well as homologous recombination activities, are detectable in mammalian mitochondrial extracts.[22,25] These activities could be potentially exploited for genome editing in mammalian mitochondria upon donor DNA delivery.

Delivery of linear DNA into isolated mammalian, including human, mitochondria has been reported.[26,27] However, DNA delivery into the mitochondria of cultured mammalian cells has been a challenge. Among possible approaches are: (1) mitochondrial targeting peptides,[28] (2) mitochondrial targeting adeno-associated viruses (AAV),[29–32] (3) fluorinated lipid nanoparticles,[33] and (4) RNA mitochondrial import signals (RMISs).[34,35] RMISs were discovered using SELEX[36] and represent hairpin RNA structures that serve as a signal to target small non-coding RNA molecules in human mitochondria.[37,38] Chimeric RNA/DNA molecules that contain RMIS and DNA sequences can be commercially synthesized. RMIS, which is based on the D-arm of yeast tRNA $^{Lys}_{CUU}$ has been reported to successfully deliver not only RNA but also short (20 nt) single-stranded DNA (ssDNA) oligonucleotides.[34,35] Nonetheless, it was

unclear whether this RMIS can be applied for the delivery of longer DNA duplexes. Here, we report the delivery of 49 nt DNA duplexes into mitochondria of cultured human cells, applying RMIS. Moreover, we demonstrate the possibility of human mitochondrial genome editing due to the delivery of ss- and double-stranded DNA (dsDNA) oligonucleotides alone or in combination with CRISPR-mito-AsCas12a into the mitochondria of human cells. Editing was carried out in the clinically relevant site of the mitochondrial *ND4* gene encompassing nucleotide G11778, whose mutations are associated with the LHON disease (Figure 1A).

RESULTS

MMEJ in human mitochondrial extracts

To confirm the presence of microhomology-mediated end joining (MMEJ) activity in human mitochondria,[22] we tested DNA end-joining activity in human mitochondrial and nuclear extracts. For this, we synthesized two oligonucleotide duplexes (Figure 1A; Table S1), bearing the same 22 nt microhomology arm, indicated as homology sequences "HS" in Figures 1A and 1C. These duplexes were incubated with mitochondrial and nuclear extracts from HEK 293T cells. Recombination between the microhomology arms of these sequences, resulting in joining their ends, indicates MMEJ activity. The recombination product is detectable by PCR (Figure 1C, MMEJ band) with primers P5 and ^{32}P-labeled P7 (Table S2). We detected MMEJ but not NHEJ activity, which is expected to give longer amplicons, in human mitochondrial extracts (Figure 1C, lanes 1 and 2), while both NHEJ and MMEJ activities could be detected in the human nuclear extract (Figure 1C, lane 3). This suggests that the detected mitochondrial MMEJ activity is not a result of nuclear contamination. The microhomology arm was purposely designed to be identical to the wild-type (WT) human mitochondrial genomic site, which is affected in LHON by the most common LHON mutation 11,778G>A[6,7] (Figure 1A), to test whether MMEJ recombination can occur at this site.

To further validate MMEJ activity in the mitochondrial lysate, we applied another test system consisting of three DNA fragments (Figures 1B and 1D; Table S1). Two DNA duplexes MMEJ-L-HS1 and MMEJ-R-HS2 harbored different sequences HS-1 and HS-2, respectively. The third one, MMEJ-linker, contained both HS-1 and HS-2 microhomology arms separated by the BamHI site. Therefore, if two MMEJ-mediated recombination events successfully occur, this will produce a DNA fragment bearing the BamHI recognition site "GGATCC," which replaces the wild-type site "GTCGCA" in the mitochondrial genome between HS-1 and HS-2 (Figures 1B and 1D). The resulting recombinant DNA was detected by PCR, and BamHI cleavage of the amplicon generated DNA fragments of the expected sizes (Figure 1D). These results suggest that the intrinsic mitochondrial MMEJ activity can be utilized for the introduction of novel DNA sequences into the human mitochondrial genome.

Delivery of dsDNA oligonucleotides into mitochondria in human cells

To validate the utility of mitochondrial MMEJ activity for mitochondrial genome editing in living human cells, we next needed to target the DNA molecules into mitochondria. Previously, RMIS has been successfully used for the delivery of artificial RNA and short (20 nt) ssDNA molecules into human mitochondria.[34–36,39,40] Here, we aimed to test whether an RNA hairpin, corresponding to the D-arm of yeast tRNA $\frac{Lys}{CUU}$ can be used to deliver longer (49 nt) dsDNA molecules, which are suitable for human mitochondrial genome editing by MMEJ. We designed chimeric oligonucleotides, which are referred to as RMIS-Dir and RMIS-Rev (Figure 2A). The 5′-RNA parts of these oligonucleotides are composed of 16 ribonucleotides, corresponding to the D-arm of yeast tRNA $\frac{Lys}{CUU}$ (in blue) where two dihydrouridine residues are substituted by uridine residues. The 3′-parts of the chimeric oligonucleotides represent 49 nt DNA sequence, corresponding to MMEJ-linker (Figure 2A). These DNA sequences contain homologous arms HS-1 and HS-2 (Figure 2A), which are required for recombination and integration in the human mitochondrial *ND4* gene. The five-nucleotide sequence at the center, together with the last nucleotide of the left flanking sequence, creates the six-nucleotide BamHI restriction site, which should replace the most common "LHON" mutation site in the mitochondrial genome. Thus, these oligonucleotides are designed to test whether the *ND4* region of the human mitochondrial genome can be edited upon their delivery into human mitochondria. The expected nucleotide substitutions would lead to the changes in the ND4 protein: Ser339Stop, Arg340Ile, and Ile341Leu (Figure 1B). Therefore, these changes in the mitochondrial genome are expected to yield a truncated *ND4* protein and impair mitochondrial respiratory function.

First, we asked whether dsDNA oligonucleotides with the RMIS on both strands (RMIS-Dir:RMIS-Rev, Figure 2C) or on only one strand (RMIS-Dir:Rev, Figure 2D) can be targeted into the mitochondria in HEK 293T cells. For this, we modified the protocol previously developed to assess the mitochondrial import of small non-coding RNAs.[36,37,41] We transfected HEK 293T cells with the chimeric molecules and analyzed nucleic acids isolated from purified mitochondria, mitoplasts (obtained by swelling of mitochondria), and mitochondria lysed by detergent (Figure 2B). To eliminate cytosolic nucleic acids, half of each fraction was treated with a mix of nucleases to ensure complete degradation of DNA duplexes outside the mitochondria (see materials and methods). Efficiency of the nuclease treatment is evident from EtBr-stained gel images (Figures 2C and 2D, bottom). Northern blot hybridization with probes to DNA sequences used for cell transfection (Dir and Rev), mitochondrial tRNA^{Thr}, and cytosolic 5.8S rRNA demonstrated that nucleic acids located within the mitochondrial matrix were protected from the nuclease cleavage (Figures 2C and 2D, probe to mt tRNA^{Thr}) while being sensitive to the nuclease treatment upon the mitochondrial membrane disruption. The 5.8S rRNA, localized in cytosolic ribosomes, was mostly sensitive to the nuclease treatment (Figures 2C and 2D). In one experiment, mitochondrial tRNA^{Thr} was degraded in mitochondria but not in mitoplasts (Figure 2C lanes 3 and 5), suggesting that mitochondria were accidently disrupted during purification. The chimeric duplexes (RMIS-Dir:RMIS-Rev and

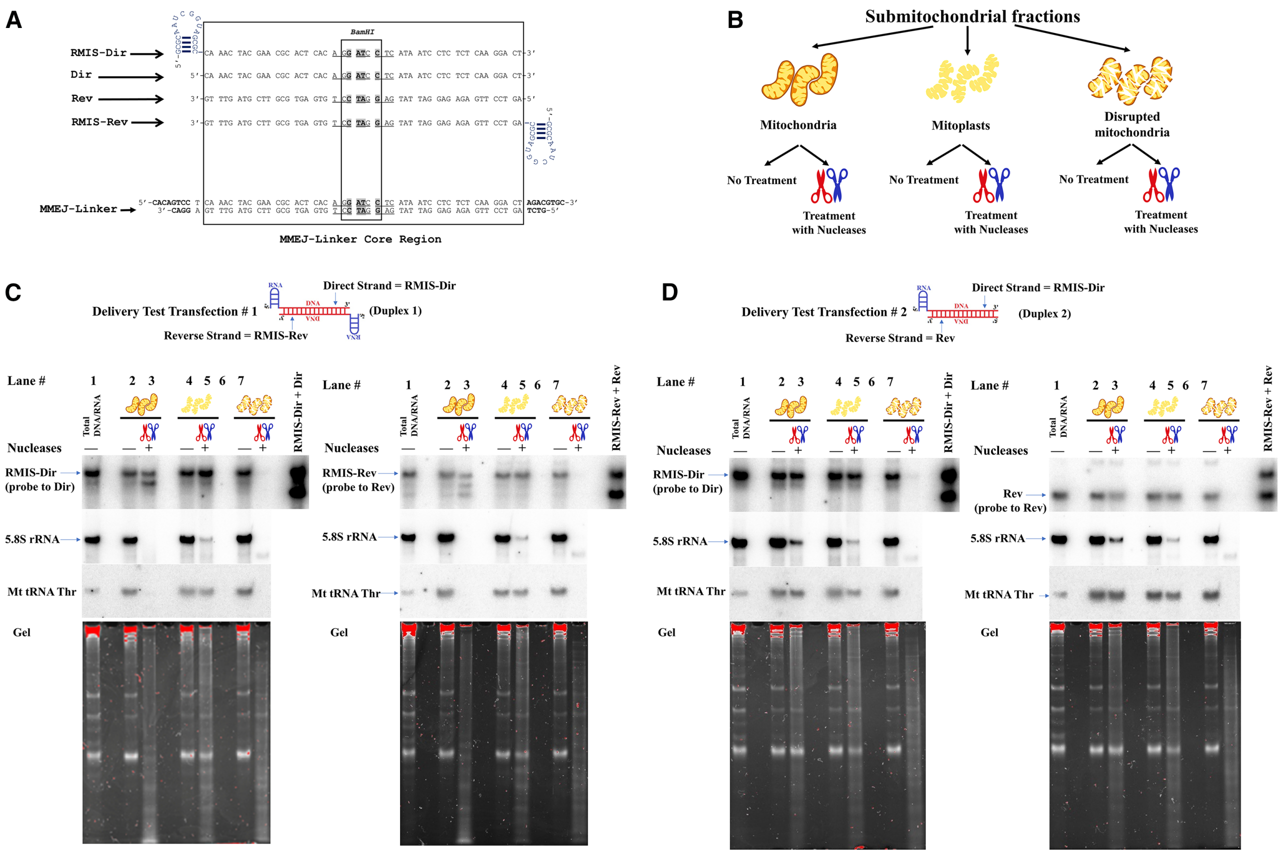

Figure 2. Evaluation of mitochondrial import of dsDNA fragments

(A) Chimeric RNA-DNA oligonucleotides RMIS-Dir, RMIS-Rev, as well as Dir and Rev, designed for the mitochondrial delivery and editing experiments, are shown in alignment to the MMEJ-linker duplex. Nucleotides that differ from the wild-type *ND4* sequence are highlighted in gray. The BamHI site is shown by the small rectangle. Nucleotides, which differ from the wild-type sequence, are highlighted in bold. The blue hairpin structures represent RMIS. (B) Schematic representation of submitochondrial fractionation (modified from Tonin et al.[34]). (C and D) *In vivo* import assays. Mitochondria were purified from the HEK 293T cells transfected with duplex 1 RMIS-Dir:RMIS-Rev (C) or with duplex 2 RMIS-Dir:Rev (D). Total nucleic acids (RNA + DNA) were purified and separated by 10% urea-PAGE for northern blot hybridization. Two representative blots and the corresponding EtBr-stained gels are shown for each experiment (left and right), probed for oligonucleotides Dir or Rev (upper left and right, correspondingly) as indicated on the left of each panel. To detect the possible cytosolic contamination, the probe for 5.8S ribosomal RNA was used (second from the top); the mitochondrial tRNA^{Thr} signal (third from the top) served as a loading control and as an indicator of mitochondrial and mitoplast integrity. The EtBr-stained gel images are shown at the bottom. Mitochondria, mitoplasts, and lysed mitochondria are schematically indicated above, as in (B). As markers of size and hybridization controls, oligonucleotides RMIS-Dir and Dir were loaded on the right extremity of each gel, as indicated on the top.

RMIS-Dir:Rev) were partially protected from the nuclease treatment in the mitoplast fractions (Figures 2C and 2D) and completely degraded by the nucleases upon lysis of mitochondria (Figures 2C and 2D).

Noteworthily, in cells transfected with duplex 1 (Figure 2C), both strands of the duplex, RMIS-Rev and RMIS-Dir, were detected in the mitoplasts upon the nuclease treatment, which indicates their delivery into the mitochondrial matrix. In cells transfected with duplex 2 (Figure 2D, left), we clearly demonstrated the mitochondrial import of the chimeric RNA-DNA strand RMIS-Dir. Remarkably, the other strand of the duplex, Rev, not harboring the RMIS signal, was also protected from the nuclease treatment in the mitochondria and mitoplast fractions (Figure 2D, right), indicating delivery of this strand into the mitochondrial matrix. These data demonstrate that a dsDNA duplex, with only one strand fused to an RNA hairpin structure, can penetrate the mitochondrial membranes, thus expanding our knowledge of the nucleic acids' mitochondrial import and their possible therapeutic applications.

Mitochondrial genome editing in human cells by donor DNA delivery

Our next objective was to test whether mitochondrial import of donor DNA fragments can induce a change in human mtDNA sequence. For this, HEK 293T cells were transfected with a mix of ssDNA oligonucleotides RMIS-Dir and RMIS-Rev or with various pre-formed duplexes (Figure 3A) capable of being targeted into the mitochondria. Six days post-transfection, mitochondria were

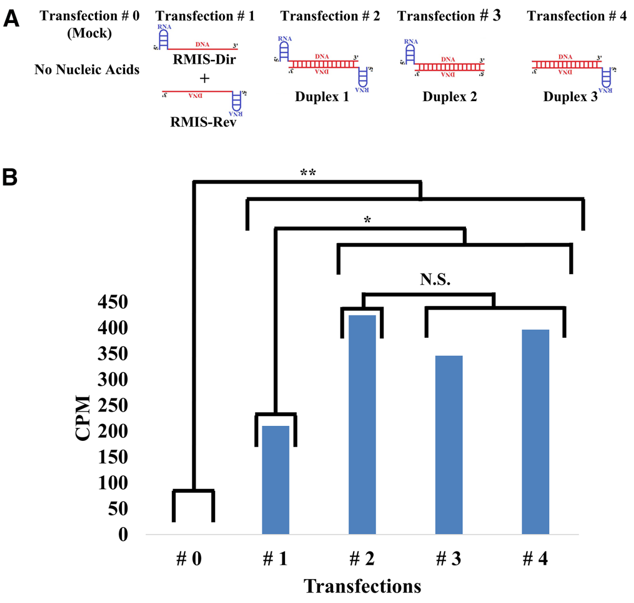

Figure 3. mtDNA editing by mitochondrially imported donor DNA in 293T cells

(A) Schematic representation of ss-oligonucleotides and duplexes used in the corresponding transfections of HEK 293T cells. Duplex 1 – RMIS-Dir:RMIS-Rev, duplex 2 – RMIS-Dir:Rev, and duplex 3 – Dir:RMIS-Rev. (B) Mitochondrial genome editing efficiency in counts of reads with the edited mitochondrial sequence per one million (CPM) of reads unambiguously aligned to the mitochondrial sequence (MT: 11,713–11,853) for each transfection is shown on the graph. Statistical significance was assessed by the two-sided permutation test with the plus-one correction and adjustment via Benjamini-Hochberg false discovery rate. N.S., no statistical significance (p adjust > 0.3). * moderate statistical significance (p adjust < 0.05; # 1 vs. # 2 – p adjust = 0.0036; # 1 vs. # 3 – p adjust = 0.043; # 1 vs. # 4 – p adjust = 0.0069). ** strong statistical significance (p adjust < 0.001).

isolated, and the targeted mtDNA region (an *ND4* gene site) was PCR-amplified and subjected to deep sequencing. Editing efficiency was defined as the number of reads containing the edited mitochondrial sequence per one million reads (counts per million [CPM]) that unambiguously aligned to the mitochondrial sequence (MT: 11710–11855). Since multiple mitochondrial DNA insertions known as nuclear mitochondrial sequences (NumtS) are found in the human nuclear genome,[42] we applied the BLAT tool of the UCSC Genome Browser to search for sequences that are similar to the sequenced amplicon in the human nuclear genome.[43] The most similar human nuclear genomic sequences (Table S3) were then aligned to the corresponding human mitochondrial genomic sequence (Figure S1A, see also materials and methods section for details). Ultimately, only those reads that clearly matched the mitochondrial but not the nuclear genome were analyzed and used for the estimation of editing efficiency (Figure 3B).

We detected a low but statistically significant amount of the expected 4 nt change (Figure 1C; Table S4) only in samples from transfected cells (Figure 3B), indicating successful microhomology-mediated editing (further referred to as MME) of the targeted gene. The mtDNA editing efficiency range was from 210 to 424 CPM (Figure 1C; Table S4). This is equivalent to the percentage of edited reads from 0.021% to 0.042% (Table S4). Notably, RMIS-bearing DNA duplexes were significantly more efficient for human mitochondrial genome editing than the combination of single-stranded RMIS-Dir and RMIS-Rev oligonucleotides (see discussion section). Thus, we succeeded in introducing the predesigned 4 nt change in the human mitochondrial genome in cultured human cells. It is important to mention that a 4 nt modification of the mitochondrial genome is currently not achievable by using other techniques, such as deaminase-based mitochondrial genome editing methods.

Next, trying to improve the efficiency of mitochondrial genome editing after donor DNA delivery, we decided to test whether mitochondrial DNA cleavage at the editing site can improve the MME efficiency. We used a recently constructed cell line, T-REx-293-Su9-AsCas12a, carrying a tetracycline-inducible gene of the mitochondria-targeted AsCas12a effector nuclease.[44] For editing, we selected a site within the mitochondrial *ND4* gene bearing the protospacer adjacent motif (PAM) TTTN required for recognition by the mito-AsCas12a effector nuclease (Figure 4A). For this experiment, new donor chimeric RNA/DNA oligonucleotides (RMIS-Dir-New and RMIS-Rev-New) were designed, each containing two different homology sequence regions (new HS-1 and new HS-2) flanking the site of specific cleavage by mito-AsCas12a-crRNA and the editing site (Figure 4A). A 5 nt change, which we expected to introduce by MME, is located in the region corresponding to crRNA; therefore, the donor DNA oligonucleotides and the edited mtDNA sequence should not be recognized and cleaved by the mito-AsCas12a-crRNA complex. Moreover, all the nucleotide changes were introduced in the third position of the codon, leading to synonymous codons and thus not inducing any alteration in the ND4 amino acid sequence.

T-REx-293-Su9-AsCas12a cells were transfected with donor ssDNA or dsDNA together with the crRNA, in combination with induction of the mito-AsCas12a with tetracycline (Figures 4B and 4C). To prevent the degradation of cleaved mtDNA molecules, some samples were also treated with an siRNA targeting mitochondrial genome maintenance exonuclease 1 (*MGME1*), as described previously.[44] MGME1 plays a key role in the degradation of linearized mtDNA; thus, its downregulation was expected to stabilize the ends of cleaved mitochondrial DNA molecules[23] and facilitate their recombination and ligation.

After cultivation of transfected cells, the level of MGME1 was checked by western blot (Figure S2), mtDNA was isolated, and the region of interest was PCR-amplified with the introduction of unique molecular identifiers (UMIs).[45] After the library preparation and deep sequencing, the editing efficiency was determined for different transfection conditions, including a control experiment without donor DNA (Figure 4C). Differences between the mitochondrial sequence and similar NumtS outside the

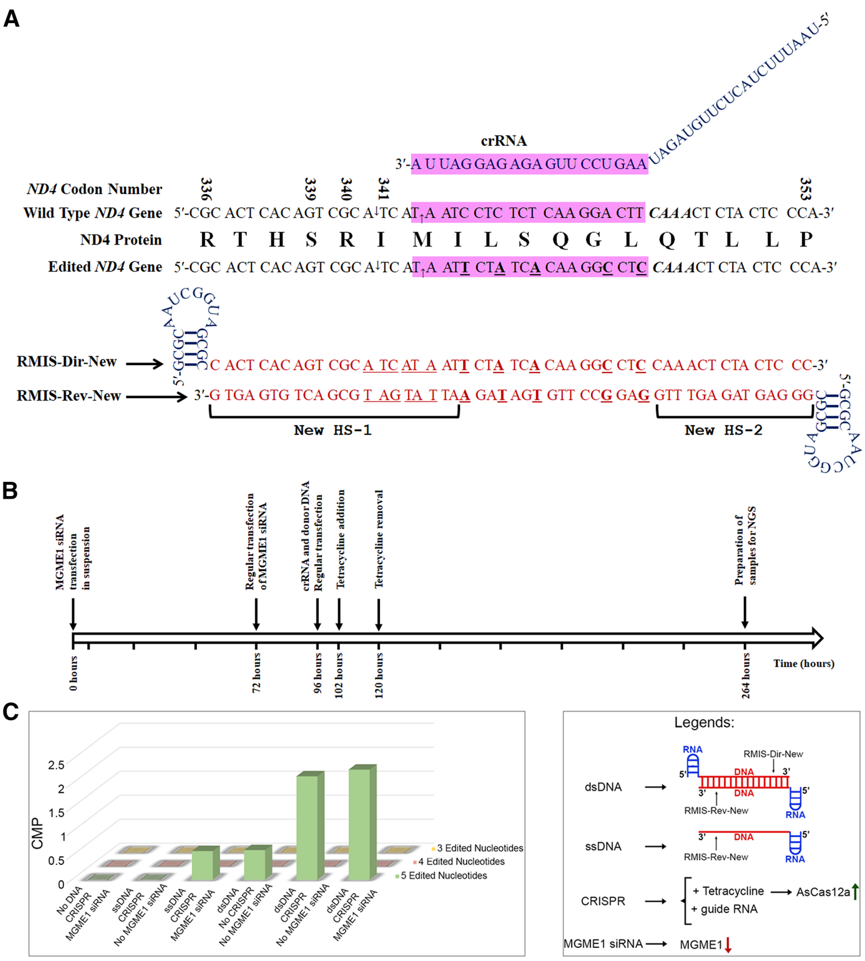

Figure 4. mtDNA editing by donor DNA delivery in the mitochondria of T-REx-293-Su9-AsCas12a cells
(A) crRNA, wild-type, and edited *ND4* alleles and their translated protein sequences, RMIS-Dir-New, and RMIS-Rev-New are shown in the picture. New HS-1 and new HS-2 indicate the microhomology arms. The guide part of crRNA and corresponding sites of wild-type and edited *ND4* genes are highlighted in purple. (B) Schematic representation of a timeline of cell transfections and treatments. (C) Left: means of counts of reads with five-, four-, or three edited nucleotides per one million (CPM, counts per million) of reads unambiguously aligned to the mitochondrial sequence (MT: 11,643–11,870) for each transfection, are shown on the 3D graph. Each transfection was conducted in biological triplicate. Right: schematic explanation of the transfections and treatments.

recombination region and the primer annealing sites (Figure S1B; Table S5) allow unambiguous alignment of reads to the mitochondrial sequence. Unexpectedly, we obtained extremely low levels of editing, from 0.7 to 2.3 CPM, which is equivalent to 7×10^{-5}%–2.3×10^{-4}% (Figure 4C; Table S6). To ensure the significance of data, we compared the numbers of reads containing the expected 5 nt change and those with 4 or 3 nucleotide changes (Figures 4C and S3; Table S6). No 4 or 3 nt changes were detected, indicating that the directed change of 5 nt was not a random event. Moreover, no editing events were found in control cells without donor DNA delivery, suggesting that the editing requires donor DNA. The 5 nt change was also not found in cells transfected with ss-donor DNA without *MGME1* downregulation, which can indicate ssDNA degradation in the mitochondria by MGME1.

The most significant level of mtDNA editing was obtained in cells transfected with ds-donor DNA and crRNA, both with and without *MGME1* silencing. Thus, as in the previous experiment, RMIS-bearing DNA duplexes were more efficient for human mitochondrial genome editing than the ss- chimeric oligonucleotides. Extremely low efficiency of editing in the last experiment may be explained by specific features of the T-REx-293-Su9-AsCas12a cell line, characterized by reduced respiration level,[44] which can influence the efficiency of cell transfection and the entire mitochondrial metabolism, including the activity of enzymes involved in the microhomology-mediated recombination.

Taken together, our results indicate that human mtDNA sequence can be modified by donor dsDNA delivery into the mitochondria. The method is currently at an early stage of development; we believe its efficiency can be improved by optimizing the structure and delivery of the donor DNA. This proof-of-concept study opens further possibilities to improve the efficiency of mtDNA editing and develop applications of this novel mtDNA editing approach.

DISCUSSION

Challenge of mtDNA editing

The objective of the study was to apply the mitochondrial machinery of MMEJ to human mtDNA editing, i.e., site-directed mutagenesis of the mitochondrial genome, a challenge that has not yet been fully resolved.

The 16.5 kb human mitochondrial genome encodes 13 transmembrane subunits of the respiratory chain complexes, 22 tRNAs, and two ribosomal RNAs required for mitochondrial translation. Therefore, any mutation in this extremely compact genome can lead to drastic changes in protein structure and/or expression, thereby causing respiratory chain dysfunction and decreased ATP production. Such defects particularly affect nervous and muscle tissue but can also be associated with autoimmune diseases, cancer, diabetes, and many other disorders.[46] Despite substantial progress in understanding the molecular mechanisms of mitochondrial diseases, there

is still a lack of treatments for patients affected by these disorders. The only notable exception is a recently approved in the European Union coenzyme Q analog, idebenone, which has a positive effect in 50% early-stage LHON patients.[47]

Recent advances in direct editing of DNA sequences offer the possibility of converting into another, thus directly correcting certain pathogenic mutations.[48] The recently reported DdCBE-based approach of mammalian mtDNA editing utilized cytosine deamination and allowed efficient conversion of C:G into T:A pairs in the tissues of living mice.[10–12] Similarly, the conversion of A:T into G:C pairs has been achieved by applying mitochondria-targeted adenosine deaminase TALEDs.[18,20,21] Besides a significant level of off-target and bystander edits[13–15] as well as context-dependence,[10] these editing approaches can introduce only transition nucleotide changes (purine-to-purine or pyrimidine-to-pyrimidine). The strategy we propose in the current study, which uses RMIS-based DNA oligonucleotide delivery and the intrinsic MMEJ enzymatic machinery for mammalian mitochondrial genome editing, is capable of introducing virtually any type of nucleotide changes, including multiple mutations in the region of interest. For this, DNA oligonucleotides bearing regions of homology with mtDNA and containing desired mutations should be delivered into the mitochondria of human cells.

DNA import into mammalian mitochondria

Here, we demonstrated that RMIS, previously discovered in our laboratory, can be used to target relatively long (~50 bp) dsDNA molecules into human mitochondria. We are aware that the experimental estimation of DNA, as well as RNA, and mitochondrial import, can be biased.[38] For this reason, we treated isolated mitochondria and mitoplasts with a mixture of nucleases, optimized to remove possible DNA contaminants from the mitochondrial surface. We also performed control experiments by lysing a portion of mitochondria before nuclease treatment; detection of DNA molecules in mitochondria and mitoplasts but not in the lysates confirmed their localization in the mitochondrial matrix (Figure 2). Interestingly, when we transfected cells with a DNA duplex carrying RMIS on a single strand, the entire duplex was detected in mitoplasts, indicating that the DNA strands were not separated during translocation across mitochondrial membranes (Figure 2D). This is consistent with our previous data, which showed that a tRNA can be imported into yeast mitochondria as a folded molecule[49] and with previous studies demonstrating the import of dsDNA in plant and human mitochondria.[26,27,50]

The molecular mechanism of nucleic acid translocation across mitochondrial membranes is not fully understood. Polynucleotide phosphorylase (PNPase), located in the intermembrane space of mitochondria, may be involved in the recognition of stem-loop RNA structures (RMIS).[51–53] Outer membrane translocase (TOM) and voltage-gated anion channel (VDAC) may facilitate translocation across the outer mitochondrial membrane (reviewed in Jeandard et al.[38]). It should be noted that VDAC oligomers have been reported to form pores for the release of mtDNA fragments,[54] and the structure and role of Porin1 hexamers have recently been studied in yeast.[55] We can thus hypothesize that pores formed by VDAC hexamers may serve not only for the export of mtDNA fragments but also for the uptake of dsDNA fragments from the cytosol.

Mitochondrial genome editing by donor DNA delivery

We demonstrated here that donor DNA delivery into human mitochondria results in a low but statistically significant introduction of designed nucleotide changes into intact mitochondrial loci (Figure 3). This donor DNA-induced editing without the preliminary cleavage of mtDNA can be explained in two ways: first, by occasional random mtDNA cleavage by reactive oxygen species (ROS)[56] or by stalling of the replication fork.[57] These events, if occurring in the region of homology with donor DNA, can induce mtDNA reparation by the MMEJ mechanism, resulting in the designed nucleotide changes which have been introduced into the donor DNA oligonucleotides. The second possibility consists of the proposed MME model. The model of the MME is shown in Figure 5. We hypothesize that RNA hairpin structures can be removed from the donor DNA molecules by mitochondrial nucleases EndoG, which digests both DNA[58,59] and RNA[59] ds-substrates with preference for C:G tracks, and ExoG, which can cleave at RNA-DNA junctions.[60] Noteworthily, the RMIS sequence has a CG track in the ds-site (Figures 2A and 4A). Implication of both nucleases in mtDNA replication and/or repair has been previously demonstrated.[59,61–63] Then, like in the MMEJ mechanism, PARP1 recruits MRE11, BRCA1, and CtIP. These proteins, performing the 5′–3′ ends recession and production of 3′ overhangs, have been identified within human mitochondria.[22] Strand invasion, microhomology alignment, and extension by DNA synthesis can then be facilitated by RAD51 and POLQ, which have both nuclear and mitochondrial localizations.[64–67] Non-homologous tails are removed by FLAP endonuclease FEN1, followed by DNA synthesis and end-joining by DNA ligase III.[57,65] Notably, FEN1 isoforms were found in human mitochondria.[68] This model is supported by our data, which demonstrate that DNA duplexes were more efficient for human mitochondrial genome editing than the single-stranded chimeric oligonucleotides (Figure 3). This can be explained not only by the rapid degradation of ssDNA but also by the fact that the ends of donor dsDNA molecules can mimic double-strand breaks in mtDNA, thereby recruiting PARP1 and the entire MMEJ machinery.

Both possible mechanisms are characterized by very low efficiency of introducing nucleotide changes (2×10^{-4}–4×10^{-4} or less). Noteworthily, these values are close to the reported efficiency of introducing changes into the intact mammalian nuclear genome of somatic cells by homologous recombination (5×10^{-4}).[69] One possible way to improve the efficiency of our approach is to introduce site-specific cleavage into mtDNA. Double-strand breaks in editing mitochondrial sites are expected to facilitate the integration of delivered oligonucleotides into these sites, enhancing the efficiency of mammalian mitochondrial genome editing. Quick degradation of linearized unedited mitochondrial genomic DNA[23,24] should further improve the efficiency of our approach. Up to date, four types

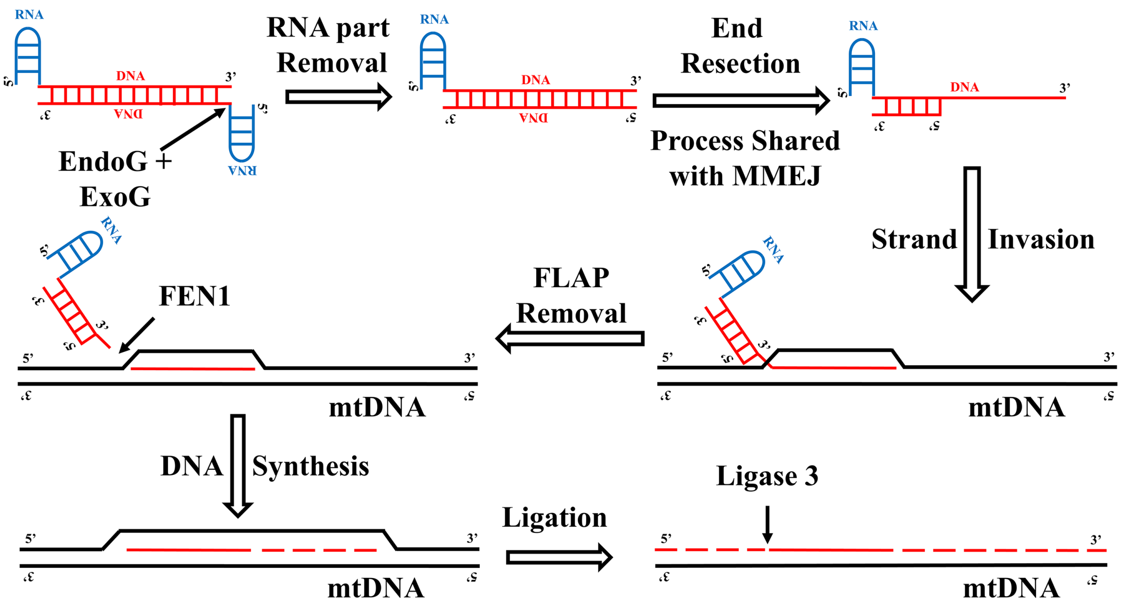

Figure 5. Hypothetical mechanism of microhomology-mediated mtDNA editing

The process may start with RMIS removal (likely mediated by EndoG and ExoG nucleases), followed by end resection, which could be executed by mitochondrial MMEJ machinery and 3′-overhangs formation. The next steps may involve strand invasion and flap removal by FEN1, followed by DNA synthesis and ligation by DNA ligase III. Newly synthesized DNA is represented by the red dashed line.

of DNA endonucleases have been successfully delivered into mitochondria and used for highly efficient site-specific mtDNA cleavage: (1) restriction enzymes (REs),[70–72] (2) zinc-finger nucleases (ZFNs),[73–76] (3) transcription activator-like effector nucleases (TALENs),[77–79] and (4) the meganuclease ARCUS.[80,81] The primary purpose of all these techniques is a heteroplasmy shift, which is supposed to improve the ratio of the wild-type to mutant mitochondrial genomic DNAs. Obviously, the heteroplasmy shift approach cannot be applied to nearly homoplasmic mitochondrial diseases, such as LHON. Nonetheless, a combination of our RMIS-based DNA oligonucleotide delivery approach with mitochondria-targeted site-specific endonucleases promises to be applicable for homoplasmic mitochondrial diseases as well. Importantly, this combined approach has the potential to introduce not only point changes into mammalian mitochondrial genomic DNA but also large deletions and insertions.

Recently, the applications of CRISPR technology for specific mitochondrial genome cleavage have been reported.[40,44] We, therefore, attempted to combine CRISPR-mito-AsCas12a technology with donor DNA delivery to improve the mitochondrial genome editing efficiency (Figure 4). A statistically significant number of the expected 5 nt change was detected in transfected cells; however, global editing was unexpectedly less efficient. This can be explained by the lower respiration capacity of the cell line and/or also by a very low efficiency of mtDNA-specific cleavage by the CRISPR-mito-AsCas12a system.[44] Notably, in these experiments, the engineered nucleotide changes were distributed over a larger region of the mitochondrial genome (16 nt) (Figure 4A), which could also significantly reduce the editing efficiency. We believe that optimization of the mitochondrial CRISPR-mito-AsCas12a system, together with donor DNA design, can create an efficient mtDNA editing tool. A very recent and elegant publication by Sifei Yin and colleagues has shown that CRISPR technology can be successfully applied to edit yeast mtDNA. In that study, the average editing frequency was 4×10^{-9}, which could be detected and quantified by yeast colony growth on selective media.[82] We believe that these and our data provide a proof of concept for the use of a CRISPR-Cas system in mitochondria, which can be further improved through ameliorated cell transfection and better RNA-targeting methodology.

In summary, we report a novel strategy of mtDNA editing in living human cells via RMIS-based DNA oligonucleotide delivery. This approach was used for the predefined change of 4 or 5 nt in the sequence of the human mitochondrial *ND4* gene. MME can introduce all possible changes into the mammalian mitochondrial genome, opening opportunities for mtDNA editing in basic and medical research.

MATERIALS AND METHODS

Cell culture

HEK293T cells (ATCC, Manassas, VA) were maintained in DMEM high glucose medium (cat # 01-052-1A, Biological Industries) complemented with 4 mM L-glutamine, 10% fetal bovine serum (FBS), and antibiotics (penicillin G 100 U/mL, streptomycin sulfate 100 μg/mL, and nystatin 12.5 U/mL). One mM pyruvate and

50 μg/mL uridine were added to the medium in order to culture HEK293T cells after mitochondrial genome editing.

Generation of the T-REx-293-Su9-AsCas12a cells with tetracycline-inducible expression of Su9-AsCas12a and culturing conditions for these cells are reported by us elsewhere.[44] When required, Su9-AsCas12a expression was induced by adding 100 ng/mL of tetracycline to the culturing medium.

Transfections

All transfections with HEK293T cells were conducted with the lipofectamine transfection reagent (cat # 18324012, ThermoFisher Scientific) according to the manufacturer's instructions. Briefly, HEK293T cells were seeded in DMEM high-glucose medium complemented with 4 mM L-glutamine, 10% FBS without any antibiotics a day before transfections. The HEK293T cells were transfected at 80%–90% confluency. The transfection mixtures of the lipofectamine reagent with oligonucleotides were prepared in DMEM high glucose medium without serum and antibiotics according to the manufacturer's instructions. An equivalent of 30 μL lipofectamine reagent and 500 pmol ss-oligonucleotide or 250 pmol oligonucleotide duplex were used per transfection in a T25 flask. Six μL of the lipofectamine reagent and 100 pmol of an ss-oligonucleotide or 50 pmol of an oligonucleotide duplex were used per transfection in a well of a 12-well plate. The medium on HEK293T cells was changed to DMEM high-glucose medium without serum and antibiotics, and the transfection mixtures were added to the cells. The medium on the transfected HEK293T cells was changed back to DMEM high-glucose medium complemented with 4 mM L-glutamine and 10% FBS, without antibiotics in 4 h after transfection. The next day after transfections, the medium on the cells was changed to the same medium with antibiotics.

The transfections of T-REx-293-Su9-AsCas12a cells were conducted as described in the subsection "mitochondrial genome editing assay with donor DNA delivery and CRISPR-Cas12a."

Mitochondria purification

Mitochondria purification for the MMEJ assay was conducted according to a shortened version of a published protocol[83] with modifications. Briefly, HEK293T cells were collected by trypsinization from 3 T75 flasks and washed with PBS. Then they were suspended in 2 mL ice-cold breakage buffer B_{cells}-1 (225 mM mannitol, 75 mM sucrose, 0.1 mM EGTA, and 30 mM Tris-HCl pH 7.4) and disrupted by one of the two methods. The cells were homogenized either with 400 strokes at 4,000 rpm applying a Dounce-type homogenizer with a Teflon pestle or by passing the cells 25–30 times through a 25-gauge needle attached to a 2 mL syringe. The homogenate was centrifuged 3 times at 600 × *g* for 5 min at +4°C in order to remove nuclei, unbroken cells, and cell debris. The mitochondria-containing supernatant was transferred to a new Eppendorf tube after each centrifugation. One-twentieth of the nuclei-containing pellet after the first centrifugation was lysed in a mitochondrial lysis buffer (50 mM Tris-HCl pH 7.5, 100 mM NaCl, 10 mM $MgCl_2$, 10% glycerol, 2 mM EGTA, 2 mM EDTA, 0.2% Triton X-100, and 1 mM DTT), complemented with half protease and phosphatase inhibitor cocktail (cat # 78441, ThermoFisher Scientific), by suspending the pellet in 100 μL ice-cold mitochondrial lysis buffer and leaving the suspension on ice for 30 min and centrifuging it at 13,200 × *g* for 5 min at +4°C. Protein concentration in the supernatant (nuclear extract) was assessed by the Bradford method, and then it was aliquoted by 10 μL, snap frozen in liquid nitrogen, and kept at −80°C. Mitochondria were precipitated from the mitochondria-containing supernatant by centrifugation at 7,000 × *g* for 10 min at +4°C. The mitochondrial pellet was suspended in 2 mL ice-cold buffer B_{cells}-2 (225 mM mannitol, 75 mM sucrose, and 30 mM Tris-HCl pH 7.4) and centrifuged again at 7,000 × *g* for 10 min at +4°C. The mitochondrial pellet was again suspended in 2 mL ice-cold buffer B_{cells}-2 and centrifuged at 10,000 × *g* for 10 min at +4°C. The resulting mitochondrial pellet was suspended in 100 μL ice-cold mitochondrial lysis buffer with half protease and phosphatase inhibitor cocktail. The lysis was conducted, and protein concentration was determined as it is described for the nuclear extract. The resulting mitochondrial extract was aliquoted by 10 μL, snap frozen in liquid nitrogen, and kept at −80°C.

Mitochondria purification from HEK293T cells for the oligonucleotide delivery assay, and deep sequencing was conducted as it was published elsewhere.[37,41] Briefly, HEK293T cells were detached and collected from one T75 flask by incubating them in 1 mM EDTA in PBS. Then, these cells were washed in PBS and suspended in 1 mL ice-cold buffer A (0.6 M sorbitol, 10 mM HEPES-KOH, pH 7.5, and 1 mM EDTA) with 0.1% BSA. The cells were disrupted by passing them 30 times through a 25-gauge needle attached to a 1 mL syringe. The homogenate was diluted 2-fold by adding 1 mL ice-cold buffer A with 0.1% BSA. The disrupted cells were then centrifuged 4 times at 600 × *g* for 5 min at +4°C in order to remove the nuclei, unbroken cells, and cell debris. The mitochondria-containing supernatant was transferred into a new Eppendorf tube each time. The mitochondria were precipitated at 15,000 × *g* for 20 min at +4°C. The mitochondrial pellet was then treated as described in subsections "oligonucleotide delivery assay" and "mitochondrial genome editing assay."

Primer and molecular size ladder labeling and radioactive PCR

Twenty pmol of P7 primer (Table S1) was labeled in 20 μL of a labeling reaction with 20 units of polynucleotide kinase T4 (PNK T4) (cat #M0201, NEB) and 13.3 pmol of ATP-[γ-^{32}P] (6,000 Ci/mmol, cat # NEG035C001MC, PerkinElmer) in the PNK T4 buffer. 0.2 pmol of the radioactively labeled P7 primer was used per 10 μL of a PCR reaction along with 5 pmol of nonradioactive P5 and 5 pmol of nonradioactive P7 primers (Table S1). The radioactive PCR was conducted with Taq polymerase at the final concentration 0.1 U/μL and at the final dNTP concentration of 0.2 pmol/μL each in a commercial Taq polymerase buffer (cat #B9004S, NEB). The PCR conditions are further specified in the subsection "MMEJ assay."

Oligonucleotides MMEJ-Dir (99 nt), MMEJ-Rev (91 nt), MMJE-linker-Dir (66 nt), MMEJ-L-Dir (54 nt), and MMEJ-L-Rev (46 nt) (Table S1) were labeled under the same conditions as the P7 primer and used as a molecular size ladder in the MMEJ assay.

MMEJ assay

The MMEJ assay was conducted as it was described by researchers from Sathees C. Raghavan's group[22,84] with modifications. Briefly, duplexes MMEJ-Ovr-L, MMEJ-Ovr-R, MMEJ-L, MMEJ-linker, and MMEJ-R were obtained by annealing of sense and antisense oligonucleotides: MMEJ-Ovr-L-Dir + MMEJ-Ovr-L-Rev, MMEJ-Ovr-R-Dir + MMEJ-Ovr-R-Rev, MMEJ-L-Dir + MMEJ-L-Rev, MMEJ-linker-Dir + MMEJ-linker-Rev, and MMEJ-R-Dir + MMEJ-R-Rev, correspondingly. The oligonucleotide sequences can be found in Table S2. Overhangs on each side of every duplex (Figures 1A and 1B) are included in order to reduce the PCR artifacts. The MMEJ assay buffer composition was 50 mM Tris-HCl pH 7.6, 20 mM $MgCl_2$, 10% PEG-3350, 1 mM ATP, and 1 mM DTT. All MMEJ reactions were conducted in a volume of 20 μL. Five μg mitochondrial or nuclear extract was added to one MMEJ reaction. In the MMEJ assays with MMEJ-Ovr-L and MMEJ-Ovr-R duplexes, each duplex was added at the final concentration of 4 nM. In the MMEJ assays with MMEJ-L, MMEJ-linker, and MMEJ-R duplexes, MMEJ-L and MMEJ-R duplexes were added in the final concentration of 4 nM each, while MMEJ-linker duplex was added at the final concentration of 8 nM. The MMEJ reactions were conducted for 4 h at 37°C and stopped by heat inactivation at 65°C for 20 min. Each MMEJ reaction was diluted 2 times with water and 2 μL was used as a template for radioactive PCR with P5 and P7 primers. The total volume of each PCR reaction was 10 μL. The PCR conditions for the MMEJ assays with MMEJ-Ovr-L and MMEJ-Ovr-R duplexes were as follows: 94°C – 3 min; cycle: 94°C – 30 s, 60°C – 1 min, and 68°C – 1 min (16 cycles); and 68°C – 5 min. The PCR conditions for the MMEJ assays with MMEJ-L, MMEJ-linker, and MMEJ-R duplexes were as follows: 94°C – 3 min; cycle: 94°C – 30 s, 60°C – 1 min, and 68°C – 1 min (26 cycles); and 68°C – 5 min.

In the case of MMEJ assays with MMEJ-Ovr-L and MMEJ-Ovr-R duplexes, 5 μL of each radioactive PCR reaction was immediately loaded and resolved on a 10% denaturing polyacrylamide gel (PAAG) with 7 M urea and 0.5× Tris-borate-EDTA (TBE). After electrophoresis, gels were dried, exposed to a phosphorimager screen, and visualized by Typhoon phosphorimager (FLA 7000).

In the case of MMEJ assays with MMEJ-L, MMEJ-linker, and MMEJ-R duplexes, we asked whether the MMEJ activity can be used to join DNA ends through a linker containing an enzyme restriction site. The introduction of a novel DNA sequence (e.g., BamHI site) is an indication of MMEJ. The design of oligonucleotide duplexes in this case was different (Figures 1B and 1D, upper right). One duplex (MMEJ-L) has a 22 nt microhomology arm, identical to the sequence located in the human mitochondrial genome to the left of the 6 nt sequence "GTCGCA" and designated "HS-1" (Figures 1B and 1D, upper right). The bold underlined G in this sequence is G11778 and is in fact the most commonly mutated nucleotide in LHON.[6,7] The other duplex has a 22 nt microhomology arm, which is designated "HS-2," identical to the sequence located in the human mitochondrial genome (to the right of the above 6 nt sequence in Figures 1B and 1D, upper at the right). These duplexes (MMEJ-L and MMEJ-R) cannot recombine with each other but only with the linker MMEJ-linker. Therefore, the radioactive PCR with primers P5 and P7 can detect a product of two MMJE-mediated recombination events with the BamHI site in the middle (Figures 1B and 1D, upper right). In order to detect this product of double recombination, the radioactive PCR reactions were diluted 2-fold with water and 2 μL of each diluted radioactive PCR reaction was digested with 20 U of BamHI (cat #R0136S, NEB) in BamHI buffer in 12 μL final volume for 6 h at 37°C. The control undigested samples were incubated for the same time at the same conditions but without BamHI. Then the samples were loaded and resolved on a 10% denaturing PAAG with 7 M urea. The gels were dried and exposed to a phosphorimager screen, which was visualized on Typhoon phosphorimager (FLA 7000).

Oligonucleotide delivery assay

Oligonucleotides, which are listed in Table S7, were used in the oligonucleotide delivery and mitochondrial genome editing assays. RMIS-Dir and RMIS-Rev hybrid oligonucleotides contain at their 5′-end a known mitochondrial RNA import signal—modified D-arm of yeast tRNA $^{Lys}_{CUU}$—rGrCrGrCrArArUrCrGrGrUrArGrCrGrC.[34,35] Duplexes 1, 2, 3, and 4 were obtained by annealing of oligonucleotides RMIS-Dir + RMIS-Rev, RMIS-Dir + Rev, Dir + RMIS-Rev, and Dir + Rev correspondingly. HEK293T cells were transfected with oligonucleotides and duplexes in T25 flasks as described in the subsection "cell culture and transfections." Immediately after transfections, the medium on the cells was changed to the medium with pyruvate and uridine. In 24 h after transfections, the transfected HEK293T cells were reseeded from T25 to T75 flasks (one T25 flask to one T75 flask). In 72 h after the transfections, mitochondria were purified from the transfected cells as described in the subsection "mitochondria purification." The purified mitochondria were divided in 6 equal parts and subjected to 10 min incubation in 100 μL of one of the following buffers: breakage buffer A (0.6 M sorbitol, 10 mM HEPES-KOH pH 7.5, and 1 mM EDTA), swelling buffer B (10 mM HEPES-KOH pH 7.5 and 1 mM EDTA), and lysing buffer C (10 mM HEPES-KOH pH 7.5, 1 mM EDTA, and 0.5% n-dodecyl-β-maltoside) with and without a mix of nucleases. The compositions of the buffers are already published elsewhere.[37,41] The mix of nucleases, which was added to each sample, consisted of 5 μL RNase A/T1 mix (2 mg/mL, 5,000 U/mL), 0.5 μL RNase I_f (50,000 units/mL), 5 μL DNase I (2,000 units/mL), 5 μL Exo I (20,000 units/mL), and 0.25 μL Exo III (100,000 units/mL). Immediately after the treatment, 1.07 mL TRI reagent (cat # 93289, Sigma-Aldrich) with adjusted pH was added to each sample. The TRI reagent pH was adjusted by adding 70 μL of 3 M KOH to each milliliter of the TRI reagent in order to ensure that the TRI reagent pH is higher than 8.0. This pH allows to purify the total nucleic acids (RNA + DNA). The total nucleic acids were then purified according to the

manufacturer protocol for RNA purification. Sixty μg of glycogen was added to the aqueous phase of each sample during the purification in order to ensure more complete oligonucleotide recovery. The purified total nucleic acids were subjected to the small RNA/DNA northern blot as it is described in the subsection "small RNA/DNA northern blot" in order to assess oligonucleotide delivery into the mitochondria.

Mitochondrial genome editing assay with donor DNA delivery only

HEK293T cells in wells of a 12-well plate were transfected with oligonucleotides and duplexes, which are described in the subsection "oligonucleotide delivery assay," as described in the subsection "cell culture and transfections." Immediately after transfections, the medium on the cells was changed to the medium with pyruvate and uridine. In 24 h after transfections, half of the transfected HEK293T cells from each well of the 12-well plate were seeded in a 35 mm cell culture dish. In 82 h after the transfections, the transfected HEK293T cells from each 35 mm dish were seeded in one T75 and one T25 flask. In 144 h after the transfections, the transfected HEK293T cells in T25 flasks were frozen, while mitochondria were purified from T75 flasks as described in the subsection "mitochondria purification." The purified mitochondria were lysed in 200 μL lysis buffer (10 mM Tris-HCl pH 8.8, 1 mM EDTA, 0.5% Triton X-100) by heating the samples at 95°C for 5 min. Two μL of each lysate was used to amplify the edited site of the human mitochondrial genome with primers Mit-ND4-DS-Dir and Mit-ND4-DS-Rev (Table S1), applying Q5 high-fidelity DNA polymerase (cat # M0491, New England Biolabs, USA). The resulting PCR products were submitted for library preparation and deep sequencing at the Genomics Applications Laboratory (Core Research Facility, Faculty of Medicine, The Hebrew University of Jerusalem, Jerusalem).

Mitochondrial genome editing assay with donor DNA delivery and CRISPR-mito-AsCas12a

For *MGME1* knockout (when required), T-REx-293-Su9-AsCas12a cells were transfected in suspension with 40 nM DsiRNA targeting *MGME1* (hs.Ri.MGME1.13.8, IDT) (Table S7) using lipofectamine RNAiMAX (Invitrogen) in Opti-MEM (Gibco, Fisher Scientific). After 6 h, the transfection medium was replaced with complete Eagle's minimal essential medium (EMEM). Three days later, transfection was repeated under standard adherent cell conditions.

After an additional 48 h, cells were transfected in suspension with crRNA (320 ng/mL) targeting the *ND4* gene of human mtDNA and donor DNA (RMIS-Rev-New or RMIS-Dir-New:RMIS-Rev-New at 2 μg/mL) using lipofectamine 2000 (Invitrogen) in Opti-MEM. After 6 h, the medium was replaced with EMEM supplemented with 100 ng/mL tetracycline to induce Su9-AsCas12a expression.

Seven days later, cells were detached, lysed, and total DNA was extracted using the QIAamp DNA Mini kit (Qiagen). Hundred ng of each purified mitochondrial DNA sample were used to amplify the edited site of the human mitochondrial genome with the introduction of UMIs according to a published protocol[45] with some modifications. Briefly, reactions of UMI introduction were conducted with the NGS-ND4-Dir primer (Table S2). Primer concentration was 0.02 μM. The total reaction volume was 10 μL. Q5 Hot Start High-Fidelity 2× Master Mix (cat. # M0494, New England Biolabs) was used for this reaction. The reaction conditions were as follows: 98°C – 2 min, 59°C – 15 min, 65°C – 15 min, and 72°C – 7 min. The reactions were purified from primers with GeneRead Size Selection kit (cat # 180514, QiaGen, Germany), according to the manufacturer's instructions. Two rounds of purification were applied. The edited site with introduced UMIs was further amplified from purified DNA with primers Ext Primer and NGS-ND4-Rev applying the Q5 Hot Start High-Fidelity 2X Master Mix (cat. # M0494, New England Biolabs). PCR conditions were as follows: 95°C – 3 min; cycle: 95°C – 30 s, 59°C – 40 s, and 68°C – 40 s (16 cycles); and 68°C – 7 min.

The obtained amplicons were submitted for library preparation and deep sequencing at the Genomics Applications Laboratory (Core Research Facility, Faculty of Medicine, The Hebrew University of Jerusalem, Jerusalem).

Small RNA/DNA northern blot

Total nucleic acid samples were resolved on denaturing 10% PAAG with 7 M urea. The transfer was conducted in 0.5× TBE at 1 mA/cm^2 on a zeta-probe membrane (cat # 1620159, Bio-Rad). After UV-crosslinking, the membranes were hybridized with the following probes: Dir and Rev (Table S7), anti-tRNA-Thr (specific to mitochondrial Thr tRNA), and anti-5-8S RNA (specific to cytosolic 5.8S rRNA) (Table S8).

Western blot

Western blot conditions as well as the antibodies used are described elsewhere.[44]

Deep sequencing

The deep sequencing of PCR products was conducted at the Genomics Applications Laboratory (Core Research Facility, Faculty of Medicine, The Hebrew University of Jerusalem, Jerusalem). Libraries were prepared according to the 16S metagenomic sequencing library preparation protocol.[85] In brief, the PCR products, purified with AMPure XP beads and indexes, which are listed in Tables S9 and S10, were introduced by 8 cycles of index PCR. Then, 150 nt single-end sequencing was conducted on the Illumina machine "NextSeq 500" (cat # SY-415-1001, Illumina) using the NextSeq 500/550 mid output kit v2.5 (150 cycles). The deep sequencing data are available in the Sequence Read Archive (SRA) database: https://www.ncbi.nlm.nih.gov/sra/PRJNA1332044.

The bioinformatic analyses of both sets of deep-sequencing data were conducted by Dr. Yuval Nevo (Info-CORE, Bioinformatics

Unit of the I-CORE at the Hebrew University of Jerusalem, Jerusalem) as outlined below.

Bioinformatic analysis of mitochondrial genome editing assay with donor DNA delivery only

Briefly, sequence quality was inspected with FastQC software. Cutadapt software[86] was employed in order to trim low-quality and adapter sequences. This software was further applied for filtering sequences shorter than 75 nt and/or lacking the forward primer sequence. Remaining low-quality reads were removed with the fastq_quality_filter software. The refined reads were further aligned to the human genome version GRCh38 employing Bowtie 2 software with default parameters.[87] Uniquely aligned reads with a single best alignment score, which span the expected amplicon mitochondrial genome positions (MT: 11,710–11,855), were then inspected for changes in the editing site. Reads with the string "T0C0G1A" in their MD tag were designated as reads with edited sequence ("Mut"). Those of the remaining reads, which had an alignment score higher than −15, were designated as reads with wild-type sequence ("WT").

The Mut reads and eight of the most similar human nuclear genomic sequences (Table S8) were then aligned to the corresponding human mitochondrial genomic sequence applying the Bowtie 2 software with permissive parameters (version 2.3.4.3, command: bowtie2 -f -L 12 –local –mp 2 –rdg 3,1 –rfg 3,1 –all -x MT -U genomic_homologues.fa). The latter provided an unambiguous verification that most of the Mut reads are indeed better aligned to the human mitochondrial genomic sequence than to any human nuclear genomic sequence. This alignment also verified that most of the Mut reads have the predesigned change in the nucleotide sequence.

Bioinformatic analysis of mitochondrial genome editing assay with donor DNA delivery and CRISPR-mito-AsCas12a

Analogous to the previous analysis, the cutadapt software was applied to trim low-quality, adapter, and poly-G sequences. The same software was utilized to remove reads, which became shorter than 95 nt after trimming of low-quality sequences. Fastq_quality_filter software was used for the removal of remaining low-quality reads. The cleaned reads were aligned to the human genome version GRCh38 employing Bowtie 2 software with the -a parameter. Uniquely aligned reads with a single best alignment score, which span the expected amplicon mitochondrial genome positions (MT: 11,790–11,803) in the expected orientation, were de-duplicated in such a way that each de-duplicated read has either a unique UMI or a unique human mitochondrial genome-aligned sequence. The unique aligned and de-duplicated reads were checked for the presence of any of the possible combinations of the 5 intended nucleotide substitutions. The uniquely aligned and de-duplicated reads with all the 5 intended nucleotide substitutions were considered as edited. The remaining reads were considered as wild-type reads.

Calculations of mtDNA editing efficiency

CPM, proportion of edited reads, and percentage of edited reads are calculated according to the formulas below.

(1) $CPM = \frac{Number\ of\ Edited\ Reads}{Total\ Number\ of\ Reads} \times 1,000,000.$

(2) Proportion of Edited Reads $= \frac{Number\ of\ Edited\ Reads}{Total\ Number\ of\ Reads}$ or

(3) Proportion of Edited Reads (by CPM) $= \frac{CPM}{10^6}$.

(4) Percentage of Edited Reads $= \frac{Number\ of\ Edited\ Reads}{Total\ Number\ of\ Reads} \times 100$ or

(5) Percentage of Edited Reads (by CPM) $= \frac{CPM}{10^6} \times 100$.

Statistics

Statistical significance of differences in the mitochondrial genome editing efficiency was assessed by the two-sided permutation test with plus-one correction and adjustment via Benjamini-Hochberg false discovery rate. One simulation with 100,000 permutations was conducted for each pair of samples.

DATA AND CODE AVAILABILITY

The deep sequencing data are available in the SRA database: https://www.ncbi.nlm.nih.gov/sra/PRJNA1332044. The raw gel autography as well as western and northern blot data are available in the Mendeley data repository: https://data.mendeley.com/datasets/vbmvks2sd4/2.

ACKNOWLEDGMENTS

We thank Dr. Yuval Nevo from Info-CORE, the I-CORE Bioinformatics Unit of the Hebrew University of Jerusalem, for Bioinformatics data analysis. We thank Anne-Marie Heckel (UMR 7156 GMGM, Strasbourg) for technical help in manipulating the cells. We thank V.V.M.'s daughter Nataliya Maximova for technical help with creating the video abstract. N.E., N.S., N.N., and I.T. were supported by the Interdisciplinary Thematic Institute IMCBio+, as part of the ITI 2021–2028 program of the University of Strasbourg, CNRS, and INSERM; IdEx Unistra (ANR-10-IDEX-0002); EUR (IMCBio ANR-17-EUR-0016); and SFRI (STRAT'US project, ANR-20-SFRI-0012) within the framework of France 2030 National Program. N.N. was supported by the Region Grand-Est (France). O.P. is supported by the German-Israeli Foundation for Scientific Research and Development (GIF; grant no. 1561) and the German-Israeli Project Cooperation (DIP; grant no. 17516). Phytor partly funded this research.

AUTHOR CONTRIBUTIONS

Research idea, experimental design, conducting experiments, data analysis, and manuscript writing and editing, V.V.M.; conducting CRISPR-related experiments, N.S.; cell line design and data analysis, N.N.; statistical analysis, R.R.; research supervision and funding acquisition, Y.M.; experimental design and manuscript editing and funding acquisition, I.T.; research supervision and funding acquisition, O.P.; and experimental design, research supervision, data analysis, and manuscript writing, N.E.

DECLARATION OF INTERESTS

Y.M. is the founder and senior consultant at Phytor. V.V.M. was an employee of Phytor. Neither the company nor any of the authors nor any immediate relatives of the authors have any patent or financial benefits related to this research.

SUPPLEMENTAL INFORMATION

Supplemental information can be found online at https://doi.org/10.1016/j.omtn.2026.102959.

A video abstract is available at https://doi.org/10.1016/j.omtn.2026.102959#mmc5.

REFERENCES

1. Smith, A.L.M., Whitehall, J.C., and Greaves, L.C. (2022). Mitochondrial DNA mutations in aging and cancer. Mol. Oncol. *16*, 3276–3294. https://doi.org/10.1002/1878-0261.13291.
2. Prasuhn, J., and Brüggemann, N. (2021). Gene Therapeutic Approaches for the Treatment of Mitochondrial Dysfunction in Parkinson's Disease. Genes (Basel) *12*. https://doi.org/10.3390/genes12111840.

3. Thompson, K., Collier, J.J., Glasgow, R.I.C., Robertson, F.M., Pyle, A., Blakely, E.L., Alston, C.L., Oláhová, M., McFarland, R., and Taylor, R.W. (2020). Recent advances in understanding the molecular genetic basis of mitochondrial disease. J. Inherit. Metab. Dis. *43*, 36–50. https://doi.org/10.1002/jimd.12104.

4. Alston, C.L., Stenton, S.L., Hudson, G., Prokisch, H., and Taylor, R.W. (2021). The genetics of mitochondrial disease: dissecting mitochondrial pathology using multiomic pipelines. J. Pathol. *254*, 430–442. https://doi.org/10.1002/path.5641.

5. McClelland, C., Meyerson, C., and Van Stavern, G. (2015). Leber hereditary optic neuropathy: current perspectives. Clin. Ophthalmol. *9*, 1165–1176. https://doi.org/10.2147/OPTH.S62021.

6. Mackey, D.A., Oostra, R.J., Rosenberg, T., Nikoskelainen, E., Bronte-Stewart, J., Poulton, J., Harding, A.E., Govan, G., Bolhuis, P.A., and Norby, S. (1996). Primary pathogenic mtDNA mutations in multigeneration pedigrees with Leber hereditary optic neuropathy. Am. J. Hum. Genet. *59*, 481–485.

7. Cui, G., Ding, H., Xu, Y., Li, B., and Wang, D.W. (2013). Applications of the method of high resolution melting analysis for diagnosis of Leber's disease and the three primary mutation spectrum of LHON in the Han Chinese population. Gene *512*, 108–112. https://doi.org/10.1016/j.gene.2012.09.110.

8. Brandt, U. (2006). Energy converting NADH:quinone oxidoreductase (complex I). Annu. Rev. Biochem. *75*, 69–92. https://doi.org/10.1146/annurev.biochem.75.103004.142539.

9. Andreazza, S., Samstag, C.L., Sanchez-Martinez, A., Fernandez-Vizarra, E., Gomez-Duran, A., Lee, J.J., Tufi, R., Hipp, M.J., Schmidt, E.K., Nicholls, T.J., et al. (2019). Mitochondrially-targeted APOBEC1 is a potent mtDNA mutator affecting mitochondrial function and organismal fitness in Drosophila. Nat. Commun. *10*, 3280. https://doi.org/10.1038/s41467-019-10857-y.

10. Mok, B.Y., de Moraes, M.H., Zeng, J., Bosch, D.E., Kotrys, A.V., Raguram, A., Hsu, F., Radey, M.C., Peterson, S.B., Mootha, V.K., et al. (2020). A bacterial cytidine deaminase toxin enables CRISPR-free mitochondrial base editing. Nature *583*, 631–637. https://doi.org/10.1038/s41586-020-2477-4.

11. Lee, H., Lee, S., Baek, G., Kim, A., Kang, B.C., Seo, H., and Kim, J.S. (2021). Mitochondrial DNA editing in mice with DddA-TALE fusion deaminases. Nat. Commun. *12*, 1190. https://doi.org/10.1038/s41467-021-21464-1.

12. Silva-Pinheiro, P., Nash, P.A., Van Haute, L., Mutti, C.D., Turner, K., and Minczuk, M. (2022). In vivo mitochondrial base editing via adeno-associated viral delivery to mouse post-mitotic tissue. Nat. Commun. *13*, 750. https://doi.org/10.1038/s41467-022-28358-w.

13. Qiu, J., Wu, H., Xie, Q., Zhou, Y., Gao, Y., Liu, J., Jiang, X., Suo, L., and Kuang, Y. (2024). Harnessing accurate mitochondrial DNA base editing mediated by DdCBEs in a predictable manner. Front. Bioeng. Biotechnol. *12*, 1372211. https://doi.org/10.3389/fbioe.2024.1372211.

14. Lei, Z., Meng, H., Liu, L., Zhao, H., Rao, X., Yan, Y., Wu, H., Liu, M., He, A., and Yi, C. (2022). Mitochondrial base editor induces substantial nuclear off-target mutations. Nature *606*, 804–811. https://doi.org/10.1038/s41586-022-04836-5.

15. Guo, J., Chen, X., Liu, Z., Sun, H., Zhou, Y., Dai, Y., Ma, Y., He, L., Qian, X., Wang, J., et al. (2022). DdCBE mediates efficient and inheritable modifications in mouse mitochondrial genome. Mol. Ther. Nucleic Acids *27*, 73–80. https://doi.org/10.1016/j.omtn.2021.11.016.

16. Mok, B.Y., Kotrys, A.V., Raguram, A., Huang, T.P., Mootha, V.K., and Liu, D.R. (2022). CRISPR-free base editors with enhanced activity and expanded targeting scope in mitochondrial and nuclear DNA. Nat. Biotechnol. *40*, 1378–1387. https://doi.org/10.1038/s41587-022-01256-8.

17. Cheng, K., Li, C., Jin, J., Qian, X., Guo, J., Shen, L., Dai, Y., Zhang, X., Li, Z., Guan, Y., et al. (2023). Engineering RsDddA as mitochondrial base editor with wide target compatibility and enhanced activity. Mol. Ther. Nucleic Acids *34*, 102028. https://doi.org/10.1016/j.omtn.2023.09.005.

18. Wei, Y., Jin, M., Huang, S., Yao, F., Ren, N., Xu, K., Li, S., Gao, P., Zhou, Y., Chen, Y., et al. (2024). Enhanced C-To-T and A-To-G Base Editing in Mitochondrial DNA with Engineered DdCBE and TALED. Adv. Sci. (Weinh). *11*, e2304113. https://doi.org/10.1002/advs.202304113.

19. Lee, S., Lee, H., Baek, G., and Kim, J.-S. (2023). Precision mitochondrial DNA editing with high-fidelity DddA-derived base editors. Nat. Biotechnol. *41*, 378–386. https://doi.org/10.1038/s41587-022-01486-w.

20. Cho, S.-I., Lee, S., Mok, Y.G., Lim, K., Lee, J., Lee, J.M., Chung, E., and Kim, J.-S. (2022). Targeted A-to-G base editing in human mitochondrial DNA with programmable deaminases. Cell *185*, 1764–1776.e12. https://doi.org/10.1016/j.cell.2022.03.039.

21. Cho, S.-I., Lim, K., Hong, S., Lee, J., Kim, A., Lim, C.J., Ryou, S., Lee, J.M., Mok, Y.G., Chung, E., et al. (2024). Engineering TALE-linked deaminases to facilitate precision adenine base editing in mitochondrial DNA. Cell *187*, 95–109.e26. https://doi.org/10.1016/j.cell.2023.11.035.

22. Tadi, S.K., Sebastian, R., Dahal, S., Babu, R.K., Choudhary, B., and Raghavan, S.C. (2016). Microhomology-mediated end joining is the principal mediator of double-strand break repair during mitochondrial DNA lesions. Mol. Biol. Cell *27*, 223–235. https://doi.org/10.1091/mbc.E15-05-0260.

23. Peeva, V., Blei, D., Trombly, G., Corsi, S., Szukszto, M.J., Rebelo-Guiomar, P., Gammage, P.A., Kudin, A.P., Becker, C., Altmüller, J., et al. (2018). Linear mitochondrial DNA is rapidly degraded by components of the replication machinery. Nat. Commun. *9*, 1727. https://doi.org/10.1038/s41467-018-04131-w.

24. Nissanka, N., Bacman, S.R., Plastini, M.J., and Moraes, C.T. (2018). The mitochondrial DNA polymerase gamma degrades linear DNA fragments precluding the formation of deletions. Nat. Commun. *9*, 2491. https://doi.org/10.1038/s41467-018-04895-1.

25. Dahal, S., Dubey, S., and Raghavan, S.C. (2018). Homologous recombination-mediated repair of DNA double-strand breaks operates in mammalian mitochondria. Cell. Mol. Life Sci. *75*, 1641–1655. https://doi.org/10.1007/s00018-017-2702-y.

26. Koulintchenko, M., Temperley, R.J., Mason, P.A., Dietrich, A., and Lightowlers, R.N. (2006). Natural competence of mammalian mitochondria allows the molecular investigation of mitochondrial gene expression. Hum. Mol. Genet. *15*, 143–154. https://doi.org/10.1093/hmg/ddi435.

27. Ibrahim, N., Handa, H., Cosset, A., Koulintchenko, M., Konstantinov, Y., Lightowlers, R.N., Dietrich, A., and Weber-Lotfi, F. (2011). DNA delivery to mitochondria: sequence specificity and energy enhancement. Pharm. Res. *28*, 2871–2882. https://doi.org/10.1007/s11095-011-0516-4.

28. Flierl, A., Jackson, C., Cottrell, B., Murdock, D., Seibel, P., and Wallace, D.C. (2003). Targeted delivery of DNA to the mitochondrial compartment via import sequence-conjugated peptide nucleic acid. Mol. Ther. *7*, 550–557. https://doi.org/10.1016/s1525-0016(03)00037-6.

29. Yu, H., Koilkonda, R.D., Chou, T.-H., Porciatti, V., Ozdemir, S.S., Chiodo, V., Boye, S.L., Boye, S.E., Hauswirth, W.W., Lewin, A.S., and Guy, J. (2012). Gene delivery to mitochondria by targeting modified adenoassociated virus suppresses Leber's hereditary optic neuropathy in a mouse model. Proc. Natl. Acad. Sci. USA *109*, E1238–E1247. https://doi.org/10.1073/pnas.1119577109.

30. Yu, H., Koilkonda, R.D., Chou, T.-H., Porciatti, V., Mehta, A., Hentall, I.D., Chiodo, V.A., Boye, S.L., Hauswirth, W.W., Lewin, A.S., and Guy, J. (2015). Consequences of zygote injection and germline transfer of mutant human mitochondrial DNA in mice. Proc. Natl. Acad. Sci. USA *112*, E5689–E5698. https://doi.org/10.1073/pnas.1506129112.

31. Yu, H., Porciatti, V., Lewin, A., Hauswirth, W., and Guy, J. (2018). Longterm Reversal of Severe Visual Loss by Mitochondrial Gene Transfer in a Mouse Model of Leber Hereditary Optic Neuropathy. Sci. Rep. *8*, 5587. https://doi.org/10.1038/s41598-018-23836-y.

32. Yu, H., Sant, D.W., Wang, G., and Guy, J. (2020). Mitochondrial Transfer of the Mutant Human ND6T14484C Gene Causes Visual Loss and Optic Neuropathy. Transl. Vis. Sci. Technol. *9*, 1. https://doi.org/10.1167/tvst.9.11.1.

33. Wang, Y., Zhao, M., Xie, H.-X., Yu, H.-Y., Yang, J.-S., Qie, L.-X., Liu, N.-H., Chen, J.-Q., Yi, Z.-J., Zhou, T.-J., et al. (2025). Mitochondria-targeted gene delivery using fluorinated lipid nanoparticles to alleviate Leber's hereditary optic neuropathy. Nat. Commun. *16*, 10891. https://doi.org/10.1038/s41467-025-65874-x.

34. Tonin, Y., Heckel, A.-M., Vysokikh, M., Dovydenko, I., Meschaninova, M., Rötig, A., Munnich, A., Venyaminova, A., Tarassov, I., and Entelis, N. (2014). Modeling of antigenomic therapy of mitochondrial diseases by mitochondrially addressed RNA targeting a pathogenic point mutation in mitochondrial DNA. J. Biol. Chem. *289*, 13323–13334. https://doi.org/10.1074/jbc.M113.528968.

35. Tonin, Y., Heckel, A.M., Dovydenko, I., Meschaninova, M., Comte, C., Venyaminova, A., Pyshnyi, D., Tarassov, I., and Entelis, N. (2014).

Characterization of chemically modified oligonucleotides targeting a pathogenic mutation in human mitochondrial DNA. Biochimie *100*, 192–199. https://doi.org/10.1016/j.biochi.2013.08.020.

36. Kolesnikova, O., Kazakova, H., Comte, C., Steinberg, S., Kamenski, P., Martin, R.P., Tarassov, I., and Entelis, N. (2010). Selection of RNA aptamers imported into yeast and human mitochondria. RNA *16*, 926–941. https://doi.org/10.1261/rna.1914110.

37. Nikitchina, N., Ulashchik, E., Shmanai, V., Heckel, A.-M., Tarassov, I., Mazunin, I., and Entelis, N. (2024). Targeting of CRISPR-Cas12a crRNAs into human mitochondria. Biochimie *217*, 74–85. https://doi.org/10.1016/j.biochi.2023.09.006.

38. Jeandard, D., Smirnova, A., Tarassov, I., Barrey, E., Smirnov, A., and Entelis, N. (2019). Import of Non-Coding RNAs into Human Mitochondria: A Critical Review and Emerging Approaches. Cells *8*, 286. https://doi.org/10.3390/cells8030286.

39. Comte, C., Tonin, Y., Heckel-Mager, A.M., Boucheham, A., Smirnov, A., Auré, K., Lombès, A., Martin, R.P., Entelis, N., and Tarassov, I. (2013). Mitochondrial targeting of recombinant RNAs modulates the level of a heteroplasmic mutation in human mitochondrial DNA associated with Kearns Sayre Syndrome. Nucleic Acids Res. *41*, 418–433. https://doi.org/10.1093/nar/gks965.

40. Loutre, R., Heckel, A.-M., Smirnova, A., Entelis, N., and Tarassov, I. (2018). Can Mitochondrial DNA be CRISPRized: Pro and Contra. IUBMB Life *70*, 1233–1239. https://doi.org/10.1002/iub.1919.

41. Jeandard, D., Smirnova, A., Fasemore, A.M., Coudray, L., Entelis, N., Förstner, K.U., Tarassov, I., and Smirnov, A. (2023). CoLoC-seq probes the global topology of organelle transcriptomes. Nucleic Acids Res. *51*, e16. https://doi.org/10.1093/nar/gkac1183.

42. Simone, D., Calabrese, F.M., Lang, M., Gasparre, G., and Attimonelli, M. (2011). The reference human nuclear mitochondrial sequences compilation validated and implemented on the UCSC genome browser. BMC Genom. *12*, 517. https://doi.org/10.1186/1471-2164-12-517.

43. Perez, G., Barber, G.P., Benet-Pages, A., Casper, J., Clawson, H., Diekhans, M., Fischer, C., Gonzalez, J.N., Hinrichs, A.S., Lee, C.M., et al. (2025). The UCSC Genome Browser database: 2025 update. Nucleic Acids Res. *53*, D1243–D1249. https://doi.org/10.1093/nar/gkae974.

44. Nikitchina, N., Heckel, A.-M., Shebanov, N., Mazunin, I., Tarassov, I., and Entelis, N. (2025). Targeted deletions in human mitochondrial DNA engineered by Type V CRISPR–Cas12a system. NAR Mol. Med. *2*, ugaf021. https://doi.org/10.1093/narmme/ugaf021.

45. Peng, Q., Vijaya Satya, R., Lewis, M., Randad, P., and Wang, Y. (2015). Reducing amplification artifacts in high multiplex amplicon sequencing by using molecular barcodes. BMC Genom. *16*, 589. https://doi.org/10.1186/s12864-015-1806-8.

46. Wallace, D.C., and Chalkia, D. (2013). Mitochondrial DNA genetics and the heteroplasmy conundrum in evolution and disease. Cold Spring Harb. Perspect. Biol. *5*, a021220. https://doi.org/10.1101/cshperspect.a021220.

47. Mei, J., Ding, P., Gao, C., Zhou, J., Li, Z., Zhang, C., and Gao, J. (2025). Mitochondrial Diseases: Molecular Pathogenesis and Therapeutic Advances. MedComm (Beijing). *6*, e70385. https://doi.org/10.1002/mco2.70385.

48. Moraes, C.T. (2024). Tools for editing the mammalian mitochondrial genome. Hum. Mol. Genet. *33*, R92–R99. https://doi.org/10.1093/hmg/ddae037.

49. Entelis, N.S., Kieffer, S., Kolesnikova, O.A., Martin, R.P., and Tarassov, I.A. (1998). Structural requirements of tRNALys for its import into yeast mitochondria. Proc. Natl. Acad. Sci. USA *95*, 2838–2843. https://doi.org/10.1073/pnas.95.6.2838.

50. Koulintchenko, M., Konstantinov, Y., and Dietrich, A. (2003). Plant mitochondria actively import DNA via the permeability transition pore complex. EMBO J. *22*, 1245–1254. https://doi.org/10.1093/emboj/cdg128.

51. Golzarroshan, B., Lin, C.-L., Li, C.-L., Yang, W.-Z., Chu, L.-Y., Agrawal, S., and Yuan, H.S. (2018). Crystal structure of dimeric human PNPase reveals why disease-linked mutants suffer from low RNA import and degradation activities. Nucleic Acids Res. *46*, 8630–8640. https://doi.org/10.1093/nar/gky642.

52. Wang, G., Chen, H.-W., Oktay, Y., Zhang, J., Allen, E.L., Smith, G.M., Fan, K.C., Hong, J.S., French, S.W., McCaffery, J.M., et al. (2010). PNPASE Regulates RNA Import into Mitochondria. Cell *142*, 456–467. https://doi.org/10.1016/j.cell.2010.06.035.

53. Vedrenne, V., Gowher, A., De Lonlay, P., Nitschke, P., Serre, V., Boddaert, N., Altuzarra, C., Mager-Heckel, A.-M., Chretien, F., Entelis, N., et al. (2012). Mutation in PNPT1 , which Encodes a Polyribonucleotide Nucleotidyltransferase, Impairs RNA Import into Mitochondria and Causes Respiratory-Chain Deficiency. Am. J. Hum. Genet. *91*, 912–918. https://doi.org/10.1016/j.ajhg.2012.09.001.

54. Kim, J., Gupta, R., Blanco, L.P., Yang, S., Shteinfer-Kuzmine, A., Wang, K., Zhu, J., Yoon, H.E., Wang, X., Kerkhofs, M., et al. (2019). VDAC oligomers form mitochondrial pores to release mtDNA fragments and promote lupus-like disease. Science *366*, 1531–1536. https://doi.org/10.1126/science.aav4011.

55. Takeda, H., Shinoda, S., Goto, C., Tsutsumi, A., Sakaue, H., Zhang, C., Hirashima, T., Konishi, Y., Ono, H., Yamamori, Y., et al. (2025). Oligomer-based functions of mitochondrial porin. Nat. Commun. *16*, 6854. https://doi.org/10.1038/s41467-025-62021-4.

56. Shokolenko, I., Venediktova, N., Bochkareva, A., Wilson, G.L., and Alexeyev, M.F. (2009). Oxidative stress induces degradation of mitochondrial DNA. Nucleic Acids Res. *37*, 2539–2548. https://doi.org/10.1093/nar/gkp100.

57. Oliveira, M.T., Pontes, C.d.B., and Ciesielski, G.L. (2020). Roles of the mitochondrial replisome in mitochondrial DNA deletion formation. Genet. Mol. Biol. *43*, e20190069. https://doi.org/10.1590/1678-4685-GMB-2019-0069.

58. Côté, J., Renaud, J., and Ruiz-Carrillo, A. (1989). Recognition of (dG)n.(dC)n sequences by endonuclease G. Characterization of the calf thymus nuclease. J. Biol. Chem. *264*, 3301–3310.

59. Côté, J., and Ruiz-Carrillo, A. (1993). Primers for mitochondrial DNA replication generated by endonuclease G. Science *261*, 765–769. https://doi.org/10.1126/science.7688144.

60. Wu, C.-C., Lin, J.L.J., Yang-Yen, H.-F., and Yuan, H.S. (2019). A unique exonuclease ExoG cleaves between RNA and DNA in mitochondrial DNA replication. Nucleic Acids Res. *47*, 5405–5419. https://doi.org/10.1093/nar/gkz241.

61. Cymerman, I.A., Chung, I., Beckmann, B.M., Bujnicki, J.M., and Meiss, G. (2008). EXOG, a novel paralog of Endonuclease G in higher eukaryotes. Nucleic Acids Res. *36*, 1369–1379. https://doi.org/10.1093/nar/gkm1169.

62. Karlowicz, A., Dubiel, A.B., Wyszkowska, M., Hossain, K.A., Czub, J., and Szymanski, M.R. (2025). Mitochondrial exonuclease EXOG supports DNA integrity by the removal of single-stranded DNA flaps. Nucleic Acids Res. *53*, gkaf099. https://doi.org/10.1093/nar/gkaf099.

63. Wiehe, R.S., Gole, B., Chatre, L., Walther, P., Calzia, E., Ricchetti, M., and Wiesmüller, L. (2018). Endonuclease G promotes mitochondrial genome cleavage and replication. Oncotarget *9*, 18309–18326. https://doi.org/10.18632/oncotarget.24822.

64. Wisnovsky, S., Sack, T., Pagliarini, D.J., Laposa, R.R., and Kelley, S.O. (2018). DNA Polymerase θ Increases Mutational Rates in Mitochondrial DNA. ACS Chem. Biol. *13*, 900–908. https://doi.org/10.1021/acschembio.8b00072.

65. Kent, T., Chandramouly, G., McDevitt, S.M., Ozdemir, A.Y., and Pomerantz, R.T. (2015). Mechanism of microhomology-mediated end-joining promoted by human DNA polymerase θ. Nat. Struct. Mol. Biol. *22*, 230–237. https://doi.org/10.1038/nsmb.2961.

66. Hastings, P.J., Ira, G., and Lupski, J.R. (2009). A microhomology-mediated break-induced replication model for the origin of human copy number variation. PLoS Genet. *5*, e1000327. https://doi.org/10.1371/journal.pgen.1000327.

67. Fontana, G.A., and Gahlon, H.L. (2020). Mechanisms of replication and repair in mitochondrial DNA deletion formation. Nucleic Acids Res. *48*, 11244–11258. https://doi.org/10.1093/nar/gkaa804.

68. Kazak, L., Reyes, A., He, J., Wood, S.R., Brea-Calvo, G., Holen, T.T., and Holt, I.J. (2013). A cryptic targeting signal creates a mitochondrial FEN1 isoform with tailed R-Loop binding properties. PLoS One *8*, e62340. https://doi.org/10.1371/journal.pone.0062340.

69. Thomas, K.R., Folger, K.R., and Capecchi, M.R. (1986). High frequency targeting of genes to specific sites in the mammalian genome. Cell *44*, 419–428. https://doi.org/10.1016/0092-8674(86)90463-0.

70. Srivastava, S., and Moraes, C.T. (2001). Manipulating mitochondrial DNA heteroplasmy by a mitochondrially targeted restriction endonuclease. Hum. Mol. Genet. *10*, 3093–3099. https://doi.org/10.1093/hmg/10.26.3093.

71. Tanaka, M., Borgeld, H.-J., Zhang, J., Muramatsu, S.i., Gong, J.-S., Yoneda, M., Maruyama, W., Naoi, M., Ibi, T., Sahashi, K., et al. (2002). Gene therapy for mitochondrial disease by delivering restriction endonuclease SmaI into mitochondria. J. Biomed. Sci. *9*, 534–541. https://doi.org/10.1159/000064726.

72. Alexeyev, M.F., Venediktova, N., Pastukh, V., Shokolenko, I., Bonilla, G., and Wilson, G.L. (2008). Selective elimination of mutant mitochondrial genomes as therapeutic strategy for the treatment of NARP and MILS syndromes. Gene Ther. *15*, 516–523. https://doi.org/10.1038/gt.2008.11.

73. Minczuk, M., Papworth, M.A., Miller, J.C., Murphy, M.P., and Klug, A. (2008). Development of a single-chain, quasi-dimeric zinc-finger nuclease for the selective degradation of mutated human mitochondrial DNA. Nucleic Acids Res. *36*, 3926–3938. https://doi.org/10.1093/nar/gkn313.

74. Minczuk, M., Kolasinska-Zwierz, P., Murphy, M.P., and Papworth, M.A. (2010). Construction and testing of engineered zinc-finger proteins for sequence-specific modification of mtDNA. Nat. Protoc. *5*, 342–356. https://doi.org/10.1038/nprot.2009.245.

75. Gammage, P.A., Rorbach, J., Vincent, A.I., Rebar, E.J., and Minczuk, M. (2014). Mitochondrially targeted ZFNs for selective degradation of pathogenic mitochondrial genomes bearing large-scale deletions or point mutations. EMBO Mol. Med. *6*, 458–466. https://doi.org/10.1002/emmm.201303672.

76. Gammage, P.A., Gaude, E., Van Haute, L., Rebelo-Guiomar, P., Jackson, C.B., Rorbach, J., Pekalski, M.L., Robinson, A.J., Charpentier, M., Concordet, J.-P., et al. (2016). Near-complete elimination of mutant mtDNA by iterative or dynamic dose-controlled treatment with mtZFNs. Nucleic Acids Res. *44*, 7804–7816. https://doi.org/10.1093/nar/gkw676.

77. Bacman, S.R., Williams, S.L., Pinto, M., Peralta, S., and Moraes, C.T. (2013). Specific elimination of mutant mitochondrial genomes in patient-derived cells by mitoTALENs. Nat. Med. *19*, 1111–1113. https://doi.org/10.1038/nm.3261.

78. Hashimoto, M., Bacman, S.R., Peralta, S., Falk, M.J., Chomyn, A., Chan, D.C., Williams, S.L., and Moraes, C.T. (2015). MitoTALEN: A General Approach to Reduce Mutant mtDNA Loads and Restore Oxidative Phosphorylation Function in Mitochondrial Diseases. Mol. Ther. *23*, 1592–1599. https://doi.org/10.1038/mt.2015.126.

79. Yang, Y., Wu, H., Kang, X., Liang, Y., Lan, T., Li, T., Tan, T., Peng, J., Zhang, Q., An, G., et al. (2018). Targeted elimination of mutant mitochondrial DNA in MELAS-iPSCs by mitoTALENs. Protein Cell *9*, 283–297. https://doi.org/10.1007/s13238-017-0499-y.

80. Zekonyte, U., Bacman, S.R., Smith, J., Shoop, W., Pereira, C.V., Tomberlin, G., Stewart, J., Jantz, D., and Moraes, C.T. (2021). Mitochondrial targeted meganuclease as a platform to eliminate mutant mtDNA in vivo. Nat. Commun. *12*, 3210. https://doi.org/10.1038/s41467-021-23561-7.

81. Shoop, W.K., Lape, J., Trum, M., Powell, A., Sevigny, E., Mischler, A., Bacman, S.R., Fontanesi, F., Smith, J., Jantz, D., et al. (2023). Efficient elimination of MELAS-associated m.3243G mutant mitochondrial DNA by an engineered mitoARCUS nuclease. Nat. Metab. *5*, 2169–2183. https://doi.org/10.1038/s42255-023-00932-6.

82. Yin, S., Jarosz, D.F., and Ting, A.Y. (2026). Towards CRISPR-based editing of the mitochondrial genome in yeast. Proc. Natl. Acad. Sci. USA *123*, e2505894123. https://doi.org/10.1073/pnas.2505894123.

83. Wieckowski, M.R., Giorgi, C., Lebiedzinska, M., Duszynski, J., and Pinton, P. (2009). Isolation of mitochondria-associated membranes and mitochondria from animal tissues and cells. Nat. Protoc. *4*, 1582–1590. https://doi.org/10.1038/nprot.2009.151.

84. Sharma, S., Javadekar, S.M., Pandey, M., Srivastava, M., Kumari, R., and Raghavan, S.C. (2015). Homology and enzymatic requirements of microhomology-dependent alternative end joining. Cell Death Dis. *6*, e1697. https://doi.org/10.1038/cddis.2015.58.

85. 16S Metagenomic Sequencing Library Preparation: Preparing 16S Ribosomal RNA Gene Amplicons for the Illumina MiSeq System https://support.illumina.com/documents/documentation/chemistry_documentation/16s/16s-metagenomic-library-prep-guide-15044223-b.pdf.

86. Martin, M. (2011). Cutadapt removes adapter sequences from high-throughput sequencing reads. EMBnet J. *17*, 10. https://doi.org/10.14806/ej.17.1.200.

87. Langmead, B., and Salzberg, S.L. (2012). Fast gapped-read alignment with Bowtie 2. Nat. Methods *9*, 357–359. https://doi.org/10.1038/nmeth.1923.

OMTN, Volume *37*

Supplemental information

Mitochondrial genome microhomology-mediated editing by donor DNA delivery into mitochondria in human cells

Vadim V. Maximov, Nikita Shebanov, Natalia Nikitchina, Rachel Rapoport, Yehoshua Maor, Ivan Tarassov, Ophry Pines, and Nina Entelis

Supplemental Material

Table S1. Oligonucleotides Used in the MMEJ Assays. Annealing sites P5 and P7 primers are underlined. The BamHI site in the oligonucleotides MMEJ-Linker-Dir and MMEJ-Linker-Rev is also underlined. Microhomology sites are in italic. All the other nucleotides are in bold.

**Oligonucleotide Name**	**Oligonucleotide Sequence**
MMEJ-L-HS-Dir	**GACT**AATGATACGGCGACCACCGA<br>*CACTCACAGTCGCATCATAATC***ATGCATAA**
MMEJ-L-HS-Rev	**GCAT***GATTATGATGCGACTGTGAGTG*<br>TCGGTGGTCGCCGTATCATT
MMEJ-R-HS-Dir	**GATTAGAT***CACTCACAGTCGCATCATAATC*<br>TCGTATGCCGTCTTCTGCTTG**TGAG**
MMEJ-R-HS-Rev	CAAGCAGAAGACGGCATACGA<br>*GATTATGATGCGACTGTGAGTG***ATCT**
MMEJ-L-HS1-Dir	**GACT**AATGATACGGCGACCACCGA<br>*TCAAACTACGAACGCACTCACA***ATGCATAA**
MMEJ-L-HS1-Rev	**GCAT***TGTGAGTGCGTTCGTAGTTTGA*<br>TCGGTGGTCGCCGTATCATT
MMEJ-Linker-Dir	**CACAGTCC***TCAAACTACGAACGCACTCACA*<br>GGATCC*TCATAATCCTCTCTCAAGGACT***AGACGTGC**
MMEJ-Linker-Rev	**GTCT***AGTCCTTGAGAGAGGATTATGA*<br>GGATCC*TGTGAGTGCGTTCGTAGTTTGA***GGAC**

MMEJ-R-HS2-Dir	**GATTAGAT***TCATAATCCTCTCTCAAGGACT*<br>TCGTATGCCGTCTTCTGCTTG**TGAG**
MMEJ-R-HS2-Rev	CAAGCAGAAGACGGCATACGA<br>*AGTCCTTGAGAGAGGATTATGA***ATCT**
MMEJ-Dir	GACTAATGATACGGCGACCACCGATCAAACTACGA<br>ACGCACTCACAGTCGCATCATAATCCTCTCTCAAG<br>GACTTCGTATGCCGTCTTCTGCTTGTGAG
MMEJ-Rev	CAAGCAGAAGACGGCATACGAAGTCCTTGAGAGAG<br>GATTATGATGCGACTGTGAGTGCGTTCGTAGTTTGA<br>TCGGTGGTCGCCGTATCATT

Table S2. Primers used in this study.

Primer Name	Primer Sequence
P5	AATGATACGGCGACCACCGA
P7	CAAGCAGAAGACGGCATACGA
Mit-ND4-DS-Dir	TCGTCGGCAGCGTCAGATGTGTATAAGAGACAG<br>CCACGGGCTTACATCCTCAT
Mit-ND4-DS-Rev	GTCTCGTGGGCTCGGAGATGTGTATAAGAGACAG<br>GCGAGGCTTGCTAGAAGTCA
Ext Pimer	TCGTCGGCAGCGTCAGAT
NGS-ND4-Dir	TCGTCGGCAGCGTCAGATGTGTATAAGAGACAG<br>NNNNNNNNNNNNGGGGTAAGGCGAGGTTAGC

NGS-ND4-Rev	GTCTCGTGGGCTCGGAGATGTGTATAAGAGACAG CCCTCGTAGTAACAGCCATTC

Table S3. Human Nuclear mitochondrial Sequences (NumtS) similar to the human mitochondrial sequence >chrM:11713-11853.

**Genomic Location 1**	**>chr5:134926943-134927083**
Genomic Sequence 1	GTGAGGCTTGCTAGAAGTCATCAAAAGGCTATTAGTGGGA GTAGGGTTTGAAGTCCTTGAGAGAGAATTATGATGCGACT GTGGGTACGTTCGTAGTTTGAGTTTGCTAGGCAGAATAGTA ATGAGGATGTAAGTCCGTGG
**Genomic Location 2**	**>chr5:100049269-100049409**
Genomic Sequence 2	GCGAGGCTTGCCAGAAGTCATCAAAAGGCTATTAGTGGGAG TAGGGTTTGAAGTCCTTGAGAGAGAATTATGATGCGGCTGT GGGTTCGCTCGTAGTTTGAGTTTGCTAGGCAGAAAAATAAG GAGGATGTAAGTCCGTGG
**Genomic Location 3**	**>chr7:64104212-64104352**
Genomic Sequence 3	GTAAGATTTGCTATAAGTCTTCAAAAGGCTATTAGTGGGAG TAGTGTTTGAAGCTCTCGAGAGAGTAATATGATTCGGCTAT GGATTCGCTCATAGTTTGAATTTGCTAGGCAGAATAGTAAG GACAAAGTAAGTCCATGG
**Genomic Location 4**	**>chr4:155455324-155455464**
Genomic Sequence 4	GTAAGATTTGCTAGAAGTCATCAAGAGGCTATTAGCGGGA

	GCAGTGTTTGAAGGCCTCAGGTAAGAAATATGGTTTGGCTA<br>TGGACTTGCTCGTAGTTTGAATTTGCTAGGCAGAAGAGTAA<br>GGATGAAGTGAGTCCATGA
**Genomic Location 5**	**>chr8:67585876-67586015**
Genomic Sequence 5	GTCTGATTTGCTAGAAGTCATCCTGAGGCTATTAGTGGGAGT<br>AGTGTTTGAAGGCCTCAGTAAGTAATATGGTTCATCTATGGG<br>CTCACTCATAGTTTGAGTTTGCTAGGCAGAATAGTAGGGAT<br>GAAGTGAGTCCATAA
**Genomic Location 6**	**>chr4:25718362-25718490**
Genomic Sequence 6	GTAAGATTTGCTAGAAGAGGCTATTAGTGGGAGCAGTGTTT<br>GAAGCCCTCGGGTAAGTAATATGGTTCATCTATGGACTCGC<br>TCATAGTTTGAATTTGCTAGGCAGAATAGTAAGGATGAAGT<br>GAATGC
**Genomic Location 7**	**>chr16:10721979-10722119**
Genomic Sequence 7	GTAAGATTTGCTAGAAGTCATCAGGAGGCTATTAGTGGAAG<br>CAATGTTTGAAGGCCTTGGAGAAGTAGTATAATTCGGCTAT<br>GGACTCGTTCATAGTTCCAATTTGCTAGGCAGAATAGTAAG<br>GATGAAGTGAGTCCATGG
**Genomic Location 8**	**>chr1:235540322-235540462**
Genomic Sequence 8	GTAAGATTTGCTAGAAGTCATCAAGAGGCTATTAGCAGAAG<br>CAGTGTTTGAAGGCGTTCGGTAAGTAATAGGGTTCACCTAT<br>GGGCCTGCTCGTAGTTTGAATTTGCTAGGCAGAATAGTAAG<br>GCTGAAGTGAGTCCATGA

Table S4. Numbers of, CPMs of, proportions of, and percentages of reads in the mitochondrial genome editing experiment with donor DNA delivery only. This table can be found in a separate Excel spreadsheet “Table S4.xlsx”.

Table S5. Human Nuclear mitochondrial Sequences (NumtS) similar to the human mitochondrial sequence >chrM:11643-11870.

**Genomic Location 1**	**>chr5:134926926-134927153**
Genomic Sequence 1	CCCTCGTAGTAACAGCCATCCTCATCCAAACCCCCTGAAGC<br>TTCACCGGCGCAGTCATTCTCATAGTCGCCCACGGACTTAC<br>ATCCTCATTACTATTCTGCCTAGCAAACTCAAACTACGAAC<br>GTACCCACAGTCGCATCATAATTCTCTCTCAAGGACTTCAAA<br>CCCTACTCCCACTAATAGCCTTTTGATGACTTCTAGCAAGCC<br>TCACTAATCTCGCCTTACCCC
**Genomic Location 2**	**>chr5:100049260-100049479**
Genomic Sequence 2	CCCTCATAGTAACAGCTATTCTCATCCAAACCCCCTGAAGCT<br>TCACCGGCGCAGTCATTCTCATAATTGCCCACGGACTTACAT<br>CCTCCTTATTTTTCTGCCTAGCAAACTCAAACTACGAGCGAA<br>CCCACAGCCGCATCATAATTCTCTCTCAAGGACTTCAAACCC<br>TACTCCCACTAATAGCCTTTTGATGACTTCTGGCAAGCCTCG<br>CCAACCTCGC
**Genomic Location 3**	**>chr7:64104195-64104401**
Genomic Sequence 3	TCATTCAAACCCCCTGAAGCTTTACTGGTGCAATTACCCTCA

	TAATTGCCCATGGACTTACTTTGTCCTTACTATTCTGCCTAG CAAATTCAAACTATGAGCGAATCCATAGCCGAATCATATTA CTCTCTCGAGAGCTTCAAACACTACTCCCACTAATAGCCTTT TGAAGACTTATAGCAAATCTTACTAACCTTGCCTTACCCC
**Genomic Location 4**	**>chr10:36434949-36435150**
Genomic Sequence 4	CAAACCCCCTGAAGCTTTACTGGTGCAATTACCCTCATAATT GCCCACAGACTTACTCTGTCCTTACTATTCTGCTTAGCAAAT TCAAATTACGAGTGAGTCCACAGCCGAATTATACTGCTCTCT TAACGCCTTCAACCATTACTTCCACTAATAGCTTTTTGATGA CTTACAGCAAATCTTACCAACCTTGCCTTACCCC

Table S6. Numbers of, CPMs of, proportions of, and percentages of reads in the mitochondrial genome editing experiment with donor DNA delivery and CRISPR/mito-AsCas12a. This table can be found in a separate Excel spreadsheet “Table S6.xlsx”.

Table S7. Oligonucleotides used in the oligonucleotide delivery and mitochondrial genome editing assays. Uppercase letters, which represent ribonucleotide residues, are always preceded by a lowercase letter “r”. All the other uppercase letters represent deoxyribonucleotide residues. Deoxyribonucleotide residues, which compose microhomology arms, are in italic. Deoxyribonucleotide residues, which differ from the human mitochondrial genome sequence, are in bold and underlined. Ribonucleotide residues of the crRNA, which are not complementary to the targeted site, are in bold.

Oligonucleotide Name	Oligonucleotide Sequence
RMIS-Dir	rGrCrGrCrArArUrCrGrGrUrArGrCrGrCCAAACTACGAA CGCACTCACAG**GAT**C**C**TCATAATCCTCTCTCAAGGACT
RMIS-Rev	rGrCrGrCrArArUrCrGrGrUrArGrCrGrCAGTCCTTGAGA GAGGATTATGA**G**G**ATC**CTGTGAGTGCGTTCGTAGTTTG
Dir	CAAACTACGAACGCACTCACAG**GAT**C**C**TCATAATCCT CTCTCAAGGACT
Rev	AGTCCTTGAGAGAGGATTATGA**G**G**ATC**CTGTGAGTGC GTTCGTAGTTTG
RMIS-Dir-New	rGrCrGrCrArArUrCrGrGrUrArGrCrGrCCACTCACAGTCG CATCATAAT**T**CT**A**TC**A**CAAGG**C**CT**C**CAAACTCTACTCCC
RMIS-Rev-New	rGrCrGrCrArArUrCrGrGrUrArGrCrGrCGGGAGTAGAGTT TG**G**AG**G**CCTTG**T**GA**T**AG**A**ATTATGATGCGACTGTGAGTG
crRNA	***rUrArArUrUrUrCrUrArCrUrCrUrUrGrUrArGrArU***rArArG rUrCrCrUrUrGrArGrArGrArGrGrArUrUrA
siRNA MGME1 Direct Strand	rGrGrGrUrGrArArArGrUrArUrGrCrUrUrUrCrCrArArGrGrCrU rUrC
siRNA MGME1 Reverse Strand	rArGrCrCrUrUrGrGrArArArGrCrArUrArCrUrUrUrCrArCrCrC

Table S8. Probes for small RNA/DNA Northern blot hybridization.

**Probe Name**	**Probe Sequence**
Anti-tRNA-Thr	TCTCCGGTTTACAAGAC
Anti-5-8S RNA	GGCCGCAAGTGCGTTCGAAG

Table S9. Description of samples for deep sequencing from the mitochondrial genome editing experiment with donor DNA only.

Sample final name	Biological description	Index1	Index2
Sample_1	Mock Transfection	AAGAGGCA	TCGCATAA
Sample_2	Transfection 1	GCTCATGA	ATAGCCTT
Sample_3	Transfection 2	ACTCGCTA	TCTTACGC
Sample_4	Transfection 3	GCGTAGTA	AGCTAGAA
Sample_5	Transfection 4	TACGCTGC	CGGAGAGA

Table S10. Description of samples for deep sequencing from the mitochondrial genome editing experiment with donor DNA and CRISPR/mito-AsCas12a.

Sample final short name	Biological condition	Index 1	Index 2
Sample_CRISPR_1-1	CRISPR + MGME1 siRNA	ACCAATTC	ACTAGAGT
Sample_CRISPR_1-2	CRISPR + MGME1 siRNA	GGTGGACG	CATGAATG
Sample_CRISPR_1-3	CRISPR + MGME1 siRNA	TGCGTCTC	GTTCATAG
Sample_CRISPR_2-1	ssDNA + CRISPR	GGATTGTG	GTGGATAT
Sample_CRISPR_2-2	ssDNA + CRISPR	GTTGCCGG	TTAAACCG
Sample_CRISPR_2-3	ssDNA + CRISPR	CGTAAAGT	GGCGTTCA
Sample_CRISPR_3-1	ssDNA + CRISPR + MGME1 siRNA	GGTGTGAA	GTACACTA
Sample_CRISPR_3-2	ssDNA + CRISPR + MGME1 siRNA	AGGTTATC	TTCTAATG
Sample_CRISPR_3-3	ssDNA + CRISPR + MGME1 siRNA	TCGATTGT	TTCCGTCA
Sample_CRISPR_4-1	dsDNA	CTAGATAA	GAGCCTTT
Sample_CRISPR_4-2	dsDNA	GGGTTCCG	CTCGAATC
Sample_CRISPR_4-3	dsDNA	AAGTAAGA	AGAGGTCA
Sample_CRISPR_5-1	dsDNA + CRISPR	TCACCCAT	TTCCCAAG

Sample_CRISPR_5-2	dsDNA + CRISPR	AATATTCG	GCTTCCAG
Sample_CRISPR_5-3	dsDNA + CRISPR	GTCCCGGA	GCATTCAC
Sample_CRISPR_6-1	dsDNA + CRISPR + MGME1 siRNA	GTGCGGTG	TGACAGTC
Sample_CRISPR_6-2	dsDNA + CRISPR + MGME1 siRNA	CCTTGACG	GTGACCTA
Sample_CRISPR_6-3	dsDNA + CRISPR + MGME1 siRNA	CTTGACGA	TGAGTTGC

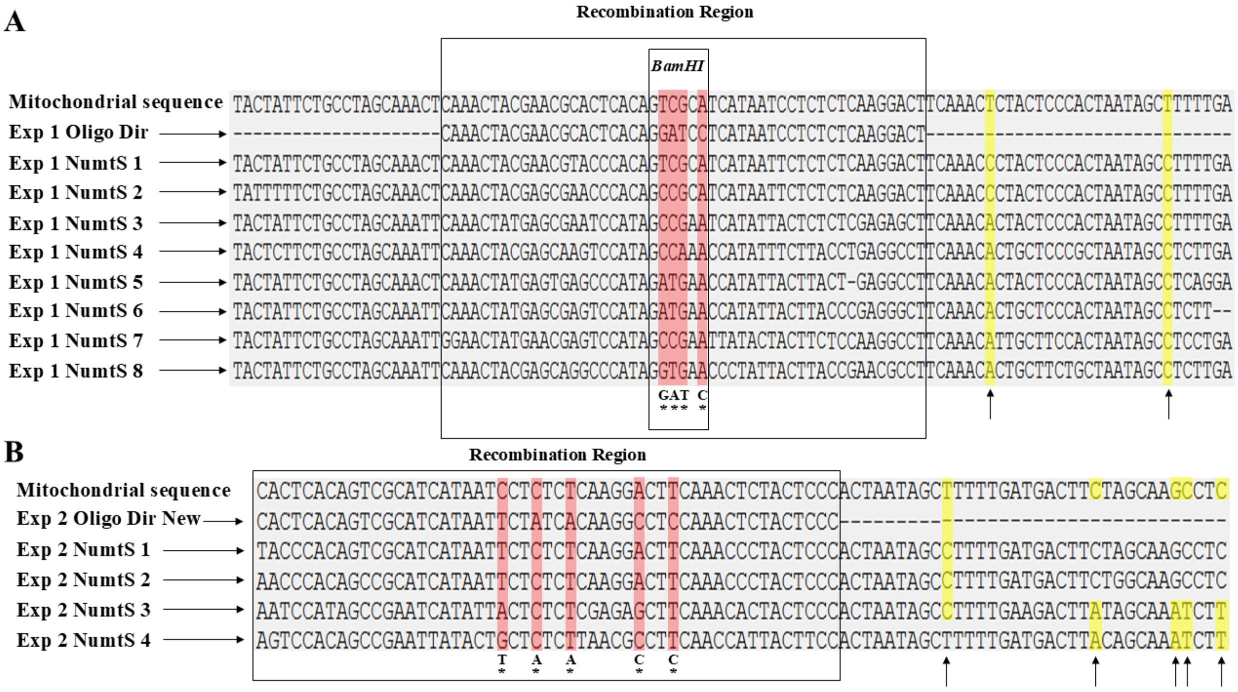

Figure S1. Alignment of the amplified human mitochondrial sequences to similar nuclear mitochondrial sequences (NumtS) at the sequenced amplicon region encompassing the edited *ND4* site. Alignments of a part the amplified and sequenced human mitochondrial genomic region,

harboring the edited *ND4* site, to the donor DNA sequences (Dir and Dir New) with the expected nucleotide edits, as well as to corresponding human NumtS, are shown. The alignments were conducted by the MAFFT software (https://mafft.cbrc.jp/alignment/server/index.html)[1,2]. Primers annealing sites for the amplified mitochondrial sequence and corresponding nuclear sequences are not included in the figure. The expected nucleotide edits are indicated at the bottom in bold and marked with asterisks. Corresponding nucleotides in the aligned sequences are highlighted in pink. The human nuclear sequences with the primers annealing sites can be found in Supplemental Tables 5 and 6. The recombination regions are surrounded by large rectangles. Nucleotide positions outside the recombination regions, at which the mitochondrial sequences differ from the corresponding nuclear sequences, are highlighted in yellow and marked by arrows at the bottom. (A) Alignment of the entire amplified and sequenced human mitochondrial genomic sequence without primers annealing sites from the experiment presented on Figure 3 to corresponding donor DNA and human NumtS is shown. The small rectangle surrounds sequence, which corresponds to the BamHI site in the edited mitochondrial DNA. (B) Alignment of the right part of the amplified and sequenced human mitochondrial genomic sequence without the primer annealing site from the experiment presented on Figure 4 to corresponding donor DNA and human NumtS is shown.

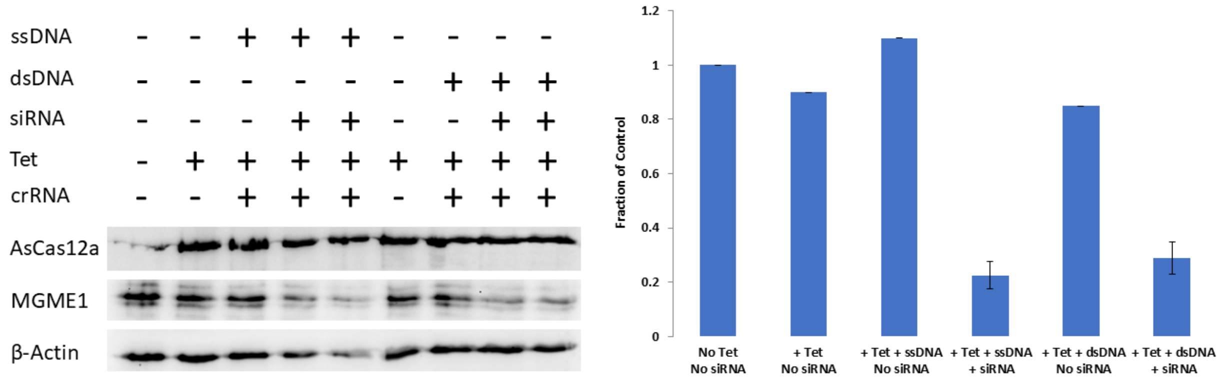

Figure S2. Western blot data on MGME1 and AsCpf1 expression under various experimental conditions. (Left panels) Western blot images are shown. (The right panel) Quantification of the MGME1 western blot bands with normalization to *β*-actin is shown. Data are presented as fraction of control without any treatment. "ssDNA" indicates transfections with the RMIS-Rev-New oligonucleotide. "dsDNA" indicates transfections with the RMIS-Dir-New:RMIS-Rev-New oligonucleotide duplex. "siRNA" indicates transfections with the *MGME1* siRNA. "Tet" indicates addition of tetracycline to the culturing medium for the *mito-AsCas12a* expression induction. "crRNA" indicates crRNA, which targets the mitochondrial *ND4* gene at the edited mtDNA site.

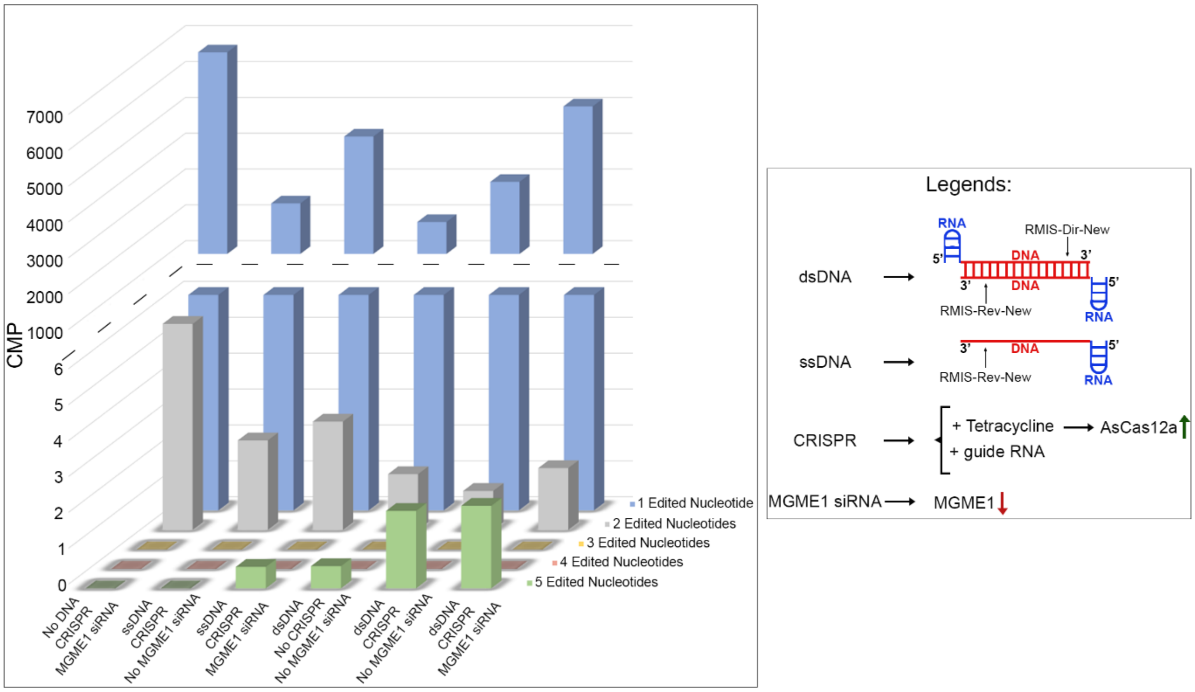

Figure S3. Introduction of changes in mtDNA by donor DNA delivery and CRISPR in mitochondria of T-REx-293-Su9-AsCas12a cells – supplemental information. Left panel: counts of reads with five-, four-, three-, two-, or one-edited nucleotides per one million (Counts Per Million = CPM) of reads unambiguously aligned to the mitochondrial sequence (MT:11643-11870) for each transfection are shown on the 3D graph. Right panel: legends, explaining transfections and treatments.

Supplemental References.

1. Katoh, K., Rozewicki, J. & Yamada, K. D. MAFFT online service: multiple sequence alignment, interactive sequence choice and visualization. *Brief Bioinform* **20**, 1160–1166 (2019).

2. Kuraku, S., Zmasek, C. M., Nishimura, O. & Katoh, K. aLeaves facilitates on-demand exploration of metazoan gene family trees on MAFFT sequence alignment server with enhanced interactivity. *Nucleic Acids Res* **41**, W22–W28 (2013).
